# SRT1720-loaded exosome-mimetic nanovesicles promote mitophagy via SIRT1/PARKIN activation to ameliorate diabetic cerebral infarction in a preclinical rat model

**DOI:** 10.1016/j.ijpx.2026.100639

**Published:** 2026-08-13

**Authors:** Qifeng Huang, Fang Zhao, Bo Sun, Xie Yang, Miao Chen

**Affiliations:** a Key Laboratory of Emergency and Trauma of Ministry of Education, Department of Emergency, The First Affiliated Hospital, Hainan Medical University, China; b Department of Radiology, The First Affiliated Hospital of Hainan Medical University, China; c Department of Emergency, Pudong Gongli Hospital, Shanghai University of Medicine & health Sciences, Shanghai, China

**Keywords:** Diabetic cerebral infarction, SRT1720, Exosome-mimetic nanovesicles, SIRT1/PARKIN pathway, Mitophagy, Neuronal protection

## Abstract

Mitochondrial dysfunction and impaired mitophagy-a key event in diabetic hyperglycemia-aggravated ischemic injury - lead to accumulation of dysfunctional mitochondria and neuronal apoptosis, which are vital processes involved in diabetic cerebral infarction (DCI). Herein, we developed SRT1720-loaded exosome-mimetic nanovesicles (SRT1720@M) to leverage the potential of SIRT1/PARKIN pathway activation to inhibit these processes and counteract DCI. The lipid fusion approach was used to fabricate exosome-loaded SRT1720@M. The properties of SRT1720@M were established by employing UV–Vis, transmission electron microscopy (TEM), dynamic light scattering, and Fourier-transform infrared (FTIR) spectroscopy. Oxygen-glucose deprivation/reperfusion (OGD/R) and high-glucose (HG)-treated primary hippocampal neurons were used as an in vitro DCI model treated with SRT1720@M. qPCR, Western blotting and immunofluorescence were used for expression analyses, whereas cell viability, phagocytosis, inflammation, and ATP/L-LA levels were assessed by CCK-8, ELISA and TEM. A rat model of DCI was obtained by establishing middle cerebral artery occlusion (MCAO), from which infarct volume, neurological deficits, histopathology, behavioral indexes, and molecular markers were detected. SRT1720@M average size was ∼68 nm, with an encapsulation efficiency of 78.19%, loading capacity of 8.52%, and zeta potential of −54 mV. In vitro, SRT1720@M markedly improved cell viability (*P* < 0.01), decreased IL-1β, TNF-α, and L-LA, but increased ATP levels, and promoted mitophagy and suppressed inflammatory responses compared with the DCI group. In the DCI model, SRT1720@M improved neurological deficits, reduced infarct volume, and alleviated behavioral disorders by inhibiting apoptosis and inflammation and promoting mitophagy and mitochondrial function through activation of the SIRT1/PARKIN pathway. SRT1720@M alleviates DCI by activating the SIRT1/PARKIN pathway and promoting mitophagy, highlighting its potential as a promising preclinical therapeutic strategy.

## 1 Introduction

Globally, stroke continues to be a major health issue, ranking second as a cause of death and third for combined death and disability. Ischemic stroke, accounting for about 80% of all strokes, remains among the leading causes of death and long-term neurological issues such as cognitive dysfunction (CD), which impacts the quality of life of patients and prognosis worldwide (Guo et al., 2023; Mao et al., 2022; Wei et al., 2025). This burden is particularly pronounced among people with Type 2 diabetes mellitus (T2DM) who experience stroke, referred to as diabetic cerebral infarction (DCI) individuals (Guo et al., 2023; Kuang et al., 2022; Mavridis et al., 2025)). Indeed, metabolic risks, particularly chronic hyperglycemia, are estimated to account for roughly 70% of deaths resulting from stroke-related disability (Guo et al., 2023; Jiang et al., 2025), and increased susceptibility to both short-term (e.g., 90-day) and long-term mortality after a stroke (Chaturvedi et al., 2020; Pantea et al., 2022; Sibon et al., 2025; Yang et al., 2021). Managing stroke in diabetes is crucial due to nearly double recurrence risk compared to non-diabetic patients (Kuang et al., 2022; McGuire et al., 2025). Despite the urgent clinical need, efficacious treatment includes robust glycemic control with blood glucose targets of 140–180 mg/dL and long-term risk management; GLP-1 receptor agonists (such as semaglutide) and pioglitazone are also crucial, given that they provide neuroprotection beyond glycemic control (Prentza et al., 2024; Suthahar, 2025). This calls for the development of novel targeted treatment schemes to efficiently monitor DCI patients.

The pathophysiology of acute ischemic stroke is characterized by immediate neural death in the focal ischemic core; however, the surrounding penumbra contains salvageable neurons that undergo delayed apoptosis (Baron, 2025; Liu et al., 2024b). A significant obstacle to effective treatment is ischemia/reperfusion (I/R) injury (IRI), in which restoring blood flow paradoxically worsens tissue damage through a massive surge in reactive oxygen species (ROS) and calcium overload (Liu et al., 2024b). Mitochondria are the primary source of this oxidative stress and are central to determining neuronal fate. Mitochondrial dysfunction caused by hyperglycemia is a hallmark of ischemic injury; mitochondrial DNA (mtDNA) enters the cytosol and acts as a potent inhibitor (Mavridis et al., 2025), leading to ROS production. While mitophagy (the removal of damaged mitochondria) restores energy after I/R, hyperglycemia in DCI patients impairs and exacerbates this process, as dysfunctional mitochondria accumulate in the brain, accelerating neuronal damage and death. Targeting mitophagy activation represents a promising therapeutic strategy for I/R injury, particularly in diabetes (Huang et al., 2025).

Sirtuin 1 (SIRT1), an NAD^+^-dependent deacetylase, controls mitochondrial biogenesis and mitophagy. Recent research shows that the SIRT1/Parkin axis protects mitochondrial health. SIRT1 deacetylates proteins to help recruit PARKIN, initiating the removal of damaged mitochondria (Huang et al., 2025). In diabetic microvascular complications, this pathway is effectively suppressed by glucose-induced stress (Sun et al., 2025a). Targeted SIRT1 activation acts as a rescue signal for the mitophagic machinery (Hu et al., 2025). Therefore, understanding how to activate this rescue in DCI patients is crucial. SRT1720, a potent synthetic activator of SIRT1, has shown promise in preclinical studies. SRT1720 decreases the Michaelis constants for acetylated substrates and NAD+ with respect to enzyme activity (Saxena et al., 2024). SRT1720 is the most specific small-molecule SIRT1 agonist (EC50 = 0.16 μM), exhibiting nearly 100-fold higher potency than resveratrol (EC50 ≈ 15 μM). Importantly, SRT1720 has been demonstrated to cross the damaged blood-brain barrier in ischemic brain injury models. In contrast, although SRT2104 also possesses SIRT1 agonist activity, data on its central nervous system distribution are insufficient, and its safety profile is less well documented (Chang et al., 2024). SRT1720@M aintains mitochondrial function by promoting mitochondrial turnover, thereby reducing neuroinflammation (Lagunas-Rangel, 2025). Nevertheless, systemic SIRT1 activators, such as SRT1720, exhibit limited blood-brain barrier (BBB) penetration and off-target effects. Advanced delivery methods are needed to circumvent these problems.

Natural exosomes have great potential but encounter significant scalability challenges. Exosome mimetics (EMS) directly address these problems through bio-interfacial engineering (Zhang et al., 2024). Exosome-mimetic nanovesicles offer three major advantages over natural exosomes and conventional nanoparticles: (1) High yield – they overcome the low production yield and complex isolation procedures of natural exosomes. (2) Tunability – their size, surface charge, and ligand modification can be flexibly controlled to optimize targeting. (3) Enhanced stability – vesicles prepared by lipid fusion exhibit prolonged in vivo circulation time and improved batch-to-batch consistency, which is critical for clinical translation (Lou et al., 2025; You et al., 2024). Compared with natural exosomes, exosome-mimetic nanovesicles prepared by lipid fusion achieve a high yield of 85–90% (natural exosomes typically <10%), with uniform particle size (PDI < 0.34) and excellent batch-to-batch reproducibility, effectively overcoming the scalability bottleneck of natural exosomes. Hybridization provides improved cargo protection and controlled-release profiles. The transition from natural to bioengineered vesicles represents a major breakthrough in nanomedicine. These bioengineered vesicles combine the biocompatibility and BBB permeability of natural exosomes with the scalability of synthetic nanoparticles, making them ideal for delivering SIRT1 activators to ischemic brain tissue (Huang et al., 2026; Khan et al., 2021). Encapsulating SRT1720 (SRT1720@M) protects it from degradation. These hybrid systems lower the risk of side effects in other organs. Bridging the gap between mitochondrial quality control and clinical stroke treatment remains a key challenge.

In this paper, we introduce SRT1720@M, a bio-inspired delivery platform capable of crossing the BBB. We hypothesize that SRT1720 promotes neuroprotection by reactivating the SIRT1/PARKIN mitophagy pathway (Sun et al., 2025a). We assessed the therapeutic efficacy of this platform in an in vivo DCI model induced by combined STZ-induced diabetes and cerebral IRI induced by middle cerebral artery occlusion (MCAO). Our findings collectively suggest that SRT1720@M is a promising strategy for treating DCI.

## 2 Materials and methods

### 2.1 Nanoparticle synthesis

The SRT1720@M delivery system was developed through a multistage biohybrid fusion process. It began with the preparation of the organic phase, in which 79 mg DSPC, 19.34 mg cholesterol, and 20 mg DSPE-mPEG2000 (AVT, Shanghai) were dissolved in 1 mL of absolute ethanol (Sinopharm). The solution was heated to 50 °C in a digital dry bath (IKA) for 15 min, after which 1 mg of SRT1720 (MCE) was added under light-protected conditions and vortexed to form a uniform, drug-loaded oil phase. After solubilization, 80 μL of the organic phase was slowly injected into 1 mL of ultrapure water using a precision syringe (Eppendorf) at 45 °C. The dispersion was stirred at 400 rpm for 30 min using a vortex mixer (Wuhan Servicebio) to promote equilibration. Polydispersity was minimized by passing the mixture 25 times through a 100 nm polycarbonate membrane using a mini-extruder (Avanti Polar Lipids). At the same time, exosomes secreted by MSCs were collected by differential ultracentrifugation at 300 *g* to 100,000 g using a Thermo Fisher Sorvall LYNX. A probe-tip sonicator (Sonics & Materials) was then used to fuse the vesicles with synthetic nanovesicles at 100 W (pulse: 2 s on/3 s off) over 5 min to allow membrane fusion without damaging exosomal surface markers.

Finally, centrifugal ultrafiltration (Millipore) was employed to remove free SRT1720 at 5000 g and 4 °C. The emergent SRT1720@M was then evaluated for granularity and yield (85–90%), and preserved in a cryogenic dewar to preserve the durable structural stability of its colloidal and pharmacological traits.

### 2.2 Encapsulation and loading efficiency determination

To ensure quantitative accuracy, a differential quantification approach was used, thereby avoiding spectral interference from vesicle components. A calibration curve was established using analytical standard SRT1720, serially diluted (1.5625–50 μg/mL), and analyzed at λ_max_ = 364 nm under a shield from ambient UV radiation. This reference curve maintained high correlation accuracy across all triplicate measurements. After using Amicon Ultra-4 filters, the filtrate and retentate volumes were measured with calibrated pipettes. The free drug fraction was diluted to fall within the spectrophotometer's linear range, and each sample was analyzed in triplicate to reduce inter-run variance (<5%). The precise weight of the cryodesiccated carrier determined the final carrier mass, accounting for buffer residues. This enabled accurate calculations of the drug-to-carrier ratio without confounding factors. The calculation formulas were:

1. Encapsulation Efficiency (EE%) = [(Total SRT1720 - Free SRT1720) / Total SRT1720] × 100.
2. Loading Capacity (LC%) = [Encapsulated SRT1720 / (Encapsulated SRT1720 + Carrier Mass)] × 100.

Calculations used the initial mass and were confirmed by the post-filtration mass balance.

### 2.3 Material characterization

#### 2.3.1 Particle size and zeta potential

The physicochemical properties of the nanoparticles were thoroughly characterized. For analysis, samples were appropriately diluted 10-fold in ultrapure deionized water (18.2 MΩ·cm) to prevent concentration-dependent agglomeration. All readings were performed at 25 °C using a Zetasizer Nano ZS90 (Malvern Panalytical) and were validated against latex beads. Particle granularity was profiled via backscatter geometry, and zeta potential was profiled by electrophoretic light scattering, against three discrete runs of 10 readings each. Stability was confirmed after 24 h at 4 °C. Results showed negligible variance (*P* > 0.05), confirming the high stability of the colloidal system.

#### 2.3.2 Drug release study

SRT1720@M was suspended in PBS (pH 7.4 or pH 5.5) and placed in a dialysis bag (MWCO 3.5 kDa). The bag was immersed in 20 mL of release medium at 37 °C under gentle shaking. At predetermined time points, aliquots were withdrawn and replaced with fresh medium. The amount of released SRT1720 was quantified by UV–Vis at 364 nm.

#### 2.3.3 TEM examination

The morphological characteristics and size distribution of the particles were examined using transmission electron microscopy (TEM). For ultrastructural analysis, 70 μL of the diluted sample was placed onto Formvar- and carbon-coated copper grids (Ted Pella). After a 3-min incubation to allow particle adherence, excess liquid was blotted off with absorbent filter paper. Contrast was enhanced by applying 70 μL of a 2% heavy-metal contrast agent to the grid for an additional 3 min. The grids were then carefully blotted dry and left to desiccate overnight at room temperature, prior to high-vacuum imaging. Visual data were documented via a FEI Tecnai G2 Spirit (120 kV). Micrographs were recorded at various magnifications (up to 50,000×) for granular structural examination. Quantitative morphometric analysis was performed using the open-source ImageJ platform. To preserve data uniformity, readings from more than 100 individual nanostructures per sample were used to establish a characteristic grain-size profile.

#### 2.3.4 UV–Vis analysis

The encapsulation and spectral integrity of SRT1720 were substantiated via UV–Vis spectrophotometry. Samples diluted in PBS were probed across 200–800 nm with a Shimadzu spectrophotometer. Spectra were acquired at 1 nm intervals to delineate absorption bands and monitor spectral changes suggestive of degradation. The raw data were ratioed to PBS to separate the formulation signal. Digital readings of peak height were taken to gauge the consistency of drug entrapment from batch to batch.

### 2.4 *FTIR* analysis

Chemical compatibility and interactions were analyzed using FTIR spectroscopy. Freeze-dried samples were mixed using a Labconco system and then pressed into pellets for spectral analysis. Spectra were acquired with a Nicolet iS5 spectrophotometer covering 400–4000 cm^−1^. Each spectrum was obtained from 32 individual scans at a consistent 4 cm^−1^ interval. The spectra were processed with standard baseline correction and normalization to investigate molecular interactions. To assess the stability and encapsulation of the formulation, results were systematically compared with spectra of the pure components to identify molecular-level interactions.

### 2.5 DCI model establishment

Male Sprague-Dawley rats (250–280 g, license SCXK(Zhe)2019–0001) were maintained in the SPF environment: the temperature of 21 ± 2C °C, the photocycle of 12:12 h, and the humidity of 50–60%. They were fed and watered freely and underwent a one-week acclimatization period to reduce animal suffering. All operations were conducted in accordance with ARRIVE guidelines and ethical standards. Randomization was done by using a computer-generated code. Health checks and body weight records were taken daily and weekly, respectively.

### 2.6 Diabetes induction

To develop a reliable model of metabolic dysfunction, experimental diabetes was induced using a freshly prepared 1% streptozotocin solution (Sigma-Aldrich) in 0.1 M citrate buffer (pH 4.5). The solution was kept in the dark to preserve potency. Subjects received a 50 mg/kg intraperitoneal injection, then a hypercaloric, lipid-rich diet for 4 weeks to induce a type 2 diabetes-like phenotype. Fasting blood glucose was measured via tail vein pricking with a calibrated enzymatic biosensor. Successful induction was defined by a fasting blood glucose level greater than 300 mg/dL at 6 h. Longitudinal monitoring was conducted weekly; any non-responders were excluded from the study to maintain cohort consistency. No exogenous insulin was administered, allowing observation of uncompensated diabetic progression.

### 2.7 MCAO establishment

Transient focal cerebral ischemia was modeled using the intraluminal filament technique. Surgical anesthesia was induced and maintained with isoflurane (5% for induction, 1.5–2% for maintenance), while core body temperature was carefully regulated at 37.0 ± 0.5 °C using a rectal probe and heating system throughout the procedure, a ventral neck midline incision allowed for the precise isolation of the extracranial branches of the carotid artery. Focal cerebral ischemia was initiated by advancing a silicone-coated monofilament through the internal carotid artery to the origin of the middle cerebral artery. After 120 min of ischemia, blood flow was restored for a 24-h recovery period. Successful occlusion and reperfusion were confirmed by real-time cortical perfusion monitoring, showing a decrease to less than 20% of baseline. Sham animals had vessel isolation without occlusion. Postoperative care included buprenorphine (0.05 mg/kg) and monitoring. To specifically model diabetic cerebral infarction (DCI) rather than standard ischemic stroke, diabetes was first induced by a single intraperitoneal injection of streptozotocin (STZ, 50 mg/kg) followed by a high-fat diet for 4 weeks. Fasting blood glucose >300 mg/dL was used to confirm successful diabetes induction. Subsequently, transient focal cerebral ischemia was induced by middle cerebral artery occlusion (MCAO) for 120 min, followed by 24 h reperfusion. To validate that this combined insult faithfully represents DCI, we included three control groups: DM alone (diabetes only, no MCAO), CIRI alone (cerebral ischemia/reperfusion injury only, no diabetes), and DM + CIRI (diabetes + MCAO). Groups (*n* = 6, based on power analysis for 80% power, α = 0.05): Sham (surgery without occlusion), Model (DCI), Model+SRT1720, Model+M, and Model+SRT1720@M (10 mg/kg/d via tail vein, 7 days post-MCAO). The sample size was calculated to ensure adequate power. Controls received PBS (vehicle). The dose was established based on pilot studies and the literature on SRT1720 safety. Post-treatment assessments (24 h after the last dose) were performed blinded. Mortality was below 10%, and replacement from reserve animals was used to maintain group size. All behavioral assessments and TTC staining analyses were performed by two independent observers who were blinded to the experimental groups, and the results were averaged. To minimize bias, behavioral tests (Y-maze, OFT, EPM, NDS) and TTC staining were conducted by two independent observers blinded to the group allocation. The data were averaged for statistical analysis.

### 2.8 In vivo brain imaging and frozen section analysis

DiR-labeled SRT1720@M was prepared by incorporating DiR (1 mg/mL) into the lipid phase during nanoparticle synthesis. DCI rats (*n* = 3 per group) were injected via the tail vein with free DiR or DiR-labeled SRT1720@M (equivalent DiR dose). At 8 h and 24 h post-injection, animals were anesthetized and imaged using an IVIS Spectrum in vivo imaging system (excitation/emission: 745/800 nm). Fluorescence signals were observed under a fluorescence microscope and quantified using ImageJ.

### 2.9 Cerebral blood flow measurement

To verify successful reduction in cerebral perfusion, real-time monitoring of cortical perfusion was performed using Laser Doppler Flowmetry (LDF) equipped with a fiber-optic microprobe. Subjects were anesthetized with a ketamine/xylazine mixture and kept warm at 37.0 ± 0.5 °C using a heating pad. The skull was then exposed after a midline scalp incision, and the connective tissue was carefully removed to improve signal quality. To monitor hemodynamic changes in the ischemic area, the measurement site was placed above the somatosensory cortex to closely observe them. Telemetry was done in the pre-occlusion period, at the moment of occlusion, and in the post-occlusion period. Signal analysis was performed using Data filters to remove light interference and normalized to a 10-min baseline preceding ischemia onset. Ischemia was established when the ultrasound Doppler showed a significant drop in perfusion immediately after the measurement. The percentage of pre-ischemic conditions was used to express Cerebral Blood Flow (CBF) and was calculated as:

CBF% = (Flux current/ Flux baseline) 100.

Animals that failed to achieve the specified occlusion level were excluded to ensure cohort consistency.

### 2.10 NDS scoring

A blinded observer assessed neurological status in a climate-controlled, quiet room 24 h after ischemia using a Zea Longa 5-point scale, based on forelimb flexion and posture stability. These were supplemented by a test of visual placement and the availability of the major primitive reflexes, namely the pinna and the startle reflexes. Deficits were classified into levels: individuals with mild hemiparesis had lower scores, whereas those with decreasing resistance to lateral movement or absence of spontaneous motor activity were rated higher on the total scale. Individual task scores were combined to form a composite Neurological Deficit Score (NDS). For the mNSS version, points were assigned for each failed task (0 = success, 1 = failure), resulting in a score typically between 0 and 18. The data were interpreted based on established preclinical neurological standards: scores of 1–6 indicated mild injury, 7–12 indicated moderate damage, and 13–18 indicated severe neurological impairment.

### 2.11 Y-maze test

To assess spatial cognition and novelty-seeking activity, the test was conducted in a 120-degree Y-maze constructed from matte-black acrylic, with three established arms (40 × 8 × 15 cm). The low illumination intensity (20 lx) helped mitigate physiological arousal. Animals were acclimated for 45 min. After each animal, the maze was cleaned and dried using a scent-neutralizing solution to remove any residual scent cues. Each animal was placed at the end of the start arm and allowed to explore the maze for 10 min. Behaviors were recorded by a high-definition sensor placed above the maze. Spatiotemporal patterns were analyzed using ANY-maze to determine mean velocity as an indicator of exploratory activity, along with the sequence of arm entries as a measure of short-term spatial working memory. Working memory performance was assessed using the Spontaneous Alternation Percentage (SAP%). An alternation was recorded when entries into three different arms occurred on successive choices. The calculation followed the standard formula: SAP% = [Number of actual alternations / (Total arm entries - 2)] × 100.

### 2.12 Elevated Plus Maze (EPM) test

The experimental setup for measuring the balance between the desire to explore and the fear of open spaces was made from medical-grade matte plastic. It included two open arms and two covered arms with 40 cm walls, extending from a central platform. The maze was placed 60 cm above the floor in a low-light testing room (around 10–15 lx). Animals were given a 45-min familiarization period in the test area before testing. Before each subject was placed on the maze surface, which was cleaned with scent-free disinfectant and dried, they were allowed 300 s to explore an open arm at the center of the junction. Spontaneous behavior was recorded with a wide-angle digital sensor directly overhead. Exploratory patterns were measured using the ANY-maze tracking software for automated behavioral analysis. The Open Arm Time Percentage (OAT%) was used to assess spatial preferences. The formula was: OAT% = [Time in open arms / (Time in open arms + Time in closed arms)] x 100.

### 2.13 Open Field Test (OFT)

The experiment was conducted in an acoustically isolated facility. A square, top-access chamber made of optically neutral polyvinyl chloride, measuring 45 × 45 × 35 cm, was deployed and provisioned with dim lighting (about 15–20 lx) conditions. Before testing, subjects got a 60-min acclimation in the testing room. To prevent olfactory cues, the arena surface was cleaned with scent-neutralizing acetic acid and dried after each trial. Each subject is then placed in the center of the field at the start of each trial. Spontaneous exploratory activity was then recorded for 15 min using a high-resolution, ceiling-mounted digital sensor interfaced with an automated tracking system. The raw video files were processed using ANY-maze to calculate specific locomotor parameters, notably the total ambulation count, as well as emotionality-related metrics, such as latency to enter the inner zone. Behavioral analysis quantified the spatial distribution of movement using the Center Time Ratio (CTR). The arena floor was digitally partitioned into a peripheral boundary and a central square (comprising the inner 25% of the total surface area). The calculation was performed as follows: CTR = (Total seconds in the central zone / Session length in seconds) × 100. A reduction in this ratio was utilized as a validated proxy for anxiogenic-like responses.

### 2.14 TTC staining

Briefly, to assess lesion volume, the brain was rapidly harvested and sectioned into 2-mm coronal sections in ice-cold neutral saline to preserve enzyme integrity. Slices were incubated in a freshly prepared 2% TTC solution (Yisheng Biotechnology, CAS 298–96-4) at body temperature for approximately 15 min, shielded from light, to facilitate enzymatic reduction to the red formazan. Following incubation, metabolically active regions appeared red, whereas infarcted zones remained pale. Subsequently, sections were fixed in 10% neutral-buffered formalin overnight to enhance contrast between zones. Digital images were acquired, and infarct size was quantified in ImageJ by summing the chromogen-deficient regions across consecutive sections to determine the total volumetric damage. Infarct volume was quantified using digital planimetry with an edema-adjusted technique to account for tissue swelling. Total volume (V) was calculated as V = Σ(Ai × T), with Ai being the corrected area of each slice and T the thickness. The final result was expressed as a percentage of the contralateral hemisphere: Infarct% % = [Volume of Contralateral Hemisphere – (Volume of Ipsilateral Hemisphere - Volume of Infarct)] / Volume of Contralateral Hemisphere × 100.

### 2.15 HE staining

In accordance with classical histological techniques, following tissue fixation in 4% paraformaldehyde and paraffin embedding, tissue sections were deparaffinized in a graded series of xylene before aqueous staining. Nuclei were stained with Mayer's hematoxylin until sufficiently blue, then differentiated in 1% acid alcohol. Subsequently, the samples were counterstained with 1% eosin for 2–3 min. Next, samples were quickly dehydrated in 95% and 100% ethanol to ensure the specimen was fully transparent before being sealed in glass. Histological features were examined under an optical microscope and assessed by two blinded pathologists to minimize observer bias.

### 2.16 Nissl staining

For Nissl staining, to examine neuronal morphology and survival, paraffin sections (5–10 μm) were deparaffinized and rehydrated to ensure optimal tissue adherence. Sections were immersed in 0.1% Cresyl Violet with gentle agitation. Afterward, they were rinsed with water and cleared in xylene for 5 min to remove residual stain. They were then mounted in DPX mounting medium to prevent dye fading due to oxidation at room temperature. Bright-field images were captured using an Olympus BX53 microscope to evaluate the density of Nissl-positive cells in the CA1 and CA3 regions.

### 2.17 TUNEL assay

To evaluate apoptosis in brain tissues, 5 μm paraffin sections of the infarct core and penumbra were deparaffinized in xylene and underwent antigen retrieval. Apoptotic DNA was labeled using a TUNEL assay kit (beyotime, C1088) following the protocol. After rinsing with PBS, we counterstained with DAPI staining solution (Beyotime, P0131) for 15 min and fluorescence images were captured using fluorescence microscope (Infinite M200) to determine the ratio of TUNEL+ cells to DAPI+ nuclei in the peri-infarct cortex.

### 2.18 qPCR

Total RNA was extracted from harvested tissues using TRIzol Reagent (Thermo Fisher Scientific, USA) according to the RNeasy Mini Kit protocols (Qiagen, Germany). To eliminate genomic DNA contamination, an on-column DNA digestion step was performed according to the manufacturer's stringent purification guidelines. RNA concentration was measured with a BioAnalyzer 2100, confirming RIN > 8.0. cDNA was synthesized from 2000 ng of template using oligo(dT) and random hexamer primers (Bio-Rad, USA) according to the manufacturer's instructions. Real-time PCR reactions were performed using SYBR Green qPCR Master Mix (TAKARA) with 0.4 μM of each primer. The program started with a 30s hot start at 95 °C, then 40 cycles of 95 °C for 10s and 60 °C for 20s on a QuantStudio 5. Non-specific products were checked with a high-resolution dissociation protocol. Data were normalized to ACTB using the 2^-ΔΔCt^ method.

**Table:** 

Genes	Forward primer sequence (5′- > 3′)	Reverse primer sequence (5′- > 3′)
SIRT1	CAACCTCCTGTTGGCTGATGA	AGCTTGCGTGTGATGCTCT
PARKIN	GCTGCTGATGATGACCTGAA	AGGTCTTGGTGATGCTGTGA
LC3	AGCAGCATCCAACCAAAATC	CTGTGTCCGTTCACCAACAG
TIM23	CTGCTGCTACTGGTGTTGGT	GCTGTAGGAGGCTGTGATGT
p62	AGGAGGAGGACCTGAAGGAG	CTGCTCTGCTGTTGGTGTAG
PINK1	AGGAGGAGGACCTGAAGGAG	CTGCTCTGCTGTTGGTGTAG
NLRP3	TGCTCTTCACTGCTATCAAGCC	ACAAGCCTTTGCTCCAGACC
Caspase-1	TGAGGCTGACAAGGAAACGA	GCTTGTTGCTGTAGGTCTGG
ACTB	AGCCATGTACGTAGCCATCC	CTCTCAGCTGTGGTGGTGAA

### 2.19 Western blot

For Western blot analysis, Total protein was extracted using RIPA lysis buffer from harvested samples, supplemented with protease and phosphatase inhibitor tablets (Beyotime, China), and then centrifuged at 12,000 *g*. Protein concentrations were determined using a microplate protein assay, and proteins were denatured by heating in SDS-PAGE sample buffer. A 12% Tris-Glycine SDS-PAGE was analyzed at a constant voltage of standardized protein samples (30 μg). The transfer of proteins was carried out at 100 V per 1 h on ice onto the activated PVDF membranes. Membranes were blocked in 5% nonfat milk in PBST buffer at room temperature for 1 h. The primary antibodies were added to membranes: Anti-SIRT1 antibody (Abcam, ab110304), LC3 polyclonal antibody (Proteintech, 14600–1-AP), Anti-p62 antibody (Abcam, ab109012), Anti-Parkin antibody (Abcam, ab77924), and Anti-PINK1 antibody (Abcam, ab186303), respectively followed by secondary antibodies Goat Anti-Rabbit IgG H&L (Abcam, Cat# 205719, 1:2000) for 1 h at room temperature. The immunoreactive signals were visualized using a SuperSignal West Pico Plus substrate and recorded on a chemiluminescence imaging system (Shanghai Qinxiang). Band intensities were measured using ImageJ and normalized to internal controls such as GAPDH. Three independent experiments were analyzed.

### 2.20 Isolation and culture of primary hippocampal neurons

To perform primary neuronal culture, Primary hippocampal neurons were dissected out of E18 Sprague-Dawley rat embryos under a stereomicroscope in the presence of ice-cold HBSS, taking care to ensure that all of the CA1-CA3 regions had been isolated. The dissociated tissues were chopped and digested with 0.25% trypsin-EDTA, pre-warmed enzymatic cocktail, to achieve maximum tissue tenderness. The tissue was subsequently digested and, in the process, mechanically dissociated using 10–15 strokes using a regular micropipette to eliminate clumps that were not dissociated, and then left to adhere in a 5% CO2 incubator at 37 °C in a controlled, humidified environment. Following pre-attachment, the medium was changed to Neurobasal with GlutaMAX and 1% antibiotic-antimycotic, and half of the medium was replaced after 3 days. To ensure a high-purity neuronal culture, 5-fluoro-2′-deoxyuridine (FUDR) was added on DIV 3 to maintain neuronal purity>95%. Neuronal health was assessed by phase-contrast microscopy after 10 days of in vitro maturation, before experimental manipulation.

### 2.21 Cell culture and OGD/R model

To establish the OGD/R model, Isolated primary hippocampal neurons were cultured in DMEM/F12 medium supplemented with 10% fetal bovine serum (FBS) in a standard incubator to maintain exponential growth. To simulate ischemic injury, cells were washed 3 times with PBS and deoxygenated before chamber placement. The cultures were then put in an anaerobic chamber with oxygen below 0.5% for 6 h. To start reoxygenation, the hypoxic buffer was replaced with fresh, oxygen-rich maintenance medium, mimicking the reperfusion phase of stroke. To simulate the diabetic condition, cells were cultured in medium containing 25 mM glucose (high glucose, HG). The combination of HG and OGD/R was used to better model the “double-hit” pathophysiological process in DCI, as chronic hyperglycemia exacerbates ischemic injury via multiple mechanisms (Oo, 2024; Simon Machado et al., 2023).

For pathway inhibition experiments, cells were treated with the selective SIRT1 inhibitor EX-527 (10 μM, Selleckchem) or PARKIN siRNA (50 nM, GenePharma) simultaneously with SRT1720@M at the onset of reoxygenation. PARKIN siRNA transfection was performed using Lipofectamine RNAiMAX (Invitrogen) according to the manufacturer's protocol. Non-targeting control siRNA was used as a negative control. Cells were harvested after 24 h for Western blot.

### 2.22 CCK-8 assay

To test cell viability, cells were taken, seeded in 96-well plate with 5 × 10^3^–1 × 10^4^ cells per well, and left to acclimatize overnight under ideal circumstances. Cells were treated with varying concentrations of the test compound in a humidified incubator at 37 °C after the adhesion period to determine the optimal therapeutic dose. Cell viability was determined by adding 10 μL of CCK-8 reagent, according to the manufacturer's instructions. The plates were then returned to a 5% CO2 incubator maintained at a controlled temperature and humidity within the linear range of the assay. OD was measured at 450 nm on a SpectraMax iD3 multi-mode reader, with background noise removed from the samples. The formula used was: [OD (treated) -OD (blank) /OD (control) -OD (blank) × 100% in three independent experimental replicates to determine the survival rate of the treated cells.

### 2.23 Cellular uptake

To evaluate the internalization of the biomimetic delivery system, FITC-tagged SRT1720@M nanoparticles were formulated by covalent FITC conjugation and by incorporating the complex into the erythrocyte membrane during formulation. Cells were incubated with labeled vesicles for 4, 8, or 12 h in a humidified environment with 5% CO2. They were then washed three times with cold PBS and fixed with 4% PFA. Nuclei were stained with DAPI at 1 μg/mL for cytoplasm reference. Confocal images were acquired using a Leica SP8 microscope with 488 nm excitation. The optical density of internalized vesicles was measured with ImageJ to evaluate uptake at each time point. For endocytosis inhibition studies, cells were pretreated with chlorpromazine (10 μg/mL, 30 min), genistein (200 μM, 30 min), amiloride (1–2 mM, 30 min), or DMSO (solvent control) at 37 °C, or incubated at 4 °C for 60 min before adding FITC-labeled SRT1720@M. After inhibitor pretreatment, cells were incubated with FITC-SRT1720@M for 4 h, washed, and analyzed by flow cytometry.

### 2.24 Mitochondrial ROS measurement

Mitochondrial superoxide levels were detected using MitoSOX Red reagent (Invitrogen). After treatments, cells were incubated with 5 μM MitoSOX in HBSS at 37 °C for 15 min, washed, and imaged by fluorescence microscopy. Fluorescence intensity was quantified using ImageJ.

### 2.25 Mitochondrial membrane potential measurement

JC-1 staining was performed using a JC-1 assay kit (Beyotime). After treatments, cells were incubated with JC-1 staining solution at 37 °C for 20 min, washed, and analyzed by fluorescence microscopy. The red/green fluorescence ratio was calculated as an indicator of mitochondrial membrane potential.

### 2.26 SIRT1 enzymatic activity test

SIRT1 enzymatic activity was measured using the SIRT1 Activity Assay Kit (Fluorometric, Abcam ab156065) according to the manufacturer‘s protocol. Briefly, equal amounts of protein (20 μg) from each group were incubated with fluorogenic substrate and NAD^+^ at 37 °C for 30 min. After adding developer solution, fluorescence intensity was monitored at Ex/Em = 350/460 nm using a microplate reader. Activity was expressed as relative fluorescence units per μg protein per minute.

### 2.27 ELISA

To measure cytokine levels, cell culture supernatants were collected, centrifuged, and stored at −80 °C. The levels of TNF-α, IL-1β, and IL-6 were determined using detection kits (Neobioscience, China) in 96-well plates. Briefly, 100 μL of standards and samples were prepared in triplicate and incubated at 37 °C for 90 min. Following a thorough washing cycle, the HRP-conjugate was incubated for 1 h at room temperature under protected light conditions. The stop solution was added using sulfuric acid, and the mixture was then spectrophotometrically read at 450 nm using a microplate reader. Quantitative measurement of absolute protein levels was performed by interpolating the standards using linear regression.

### 2.28 Immunofluorescence

To assess in situ protein expression following the treatments, the cell monolayers were fixed in 4% paraformaldehyde in PBS for 15 min at room temperature. They were then permeabilized with 0.1% Triton X-100 and blocked with a protein blocker to reduce background. Primary antibodies to MAP2, NeuN, or other proteins were incubated with gentle agitation after three five-minute washes, and secondary antibodies conjugated to either the FITC or Cy3 fluorophores were added and incubated for 1 h at room temperature. The nuclei were then counterstained with DAPI at 1 μg/mL to provide a structural reference. Images acquired by fluorescence microscopy were obtained with a Zeiss LSM 980 confocal microscope equipped with a 63× oil-immersion objective and analyzed in ImageJ.

### 2.29 Ultrastructural Transmission Electron Microscopy (TEM)

Cell pellets were collected by centrifugation and fixed in 2.5% glutaraldehyde at 4 °C for at least 4 h. Post-fixation in 1% osmium tetroxide, the samples were filled with acetone, embedded in Araldite resin, and cured for 48 h. A microtome was used to section resin blocks at 70 nm using a diamond knife, and the sections were placed on 200-mesh copper grids. The grids were double-stained with uranyl acetate and lead citrate for 15 min each at room temperature. Ultrastructural features were observed using a JEM-1400 Flash transmission electron microscope at an accelerating voltage of 80 kV to evaluate mitochondrial health and autophagic vacuoles.

### 2.30 Statistical analysis

Data processing was executed using GraphPad Prism 10.0. Before analysis, data normality was checked with the Shapiro-Wilk test, and equality of variances was tested using Levene's test. Data that were normally distributed were reported as mean ± SD from at least three independent experiments. When comparing two experimental sets, a two-tailed independent *t*-test was applied, with Welch's correction applied if variances were unequal. To compare differences between 3 or more groups, we used one-way ANOVA with Dunnett's test to identify specific group differences. A *p*-value below 0.05 (two-tailed) was considered significant. Significance levels: *p < 0.05, **p < 0.01, ***p < 0.001.

## 3 Results

### 3.1 Characterization of SRT1720@M

**Fig. 1:**
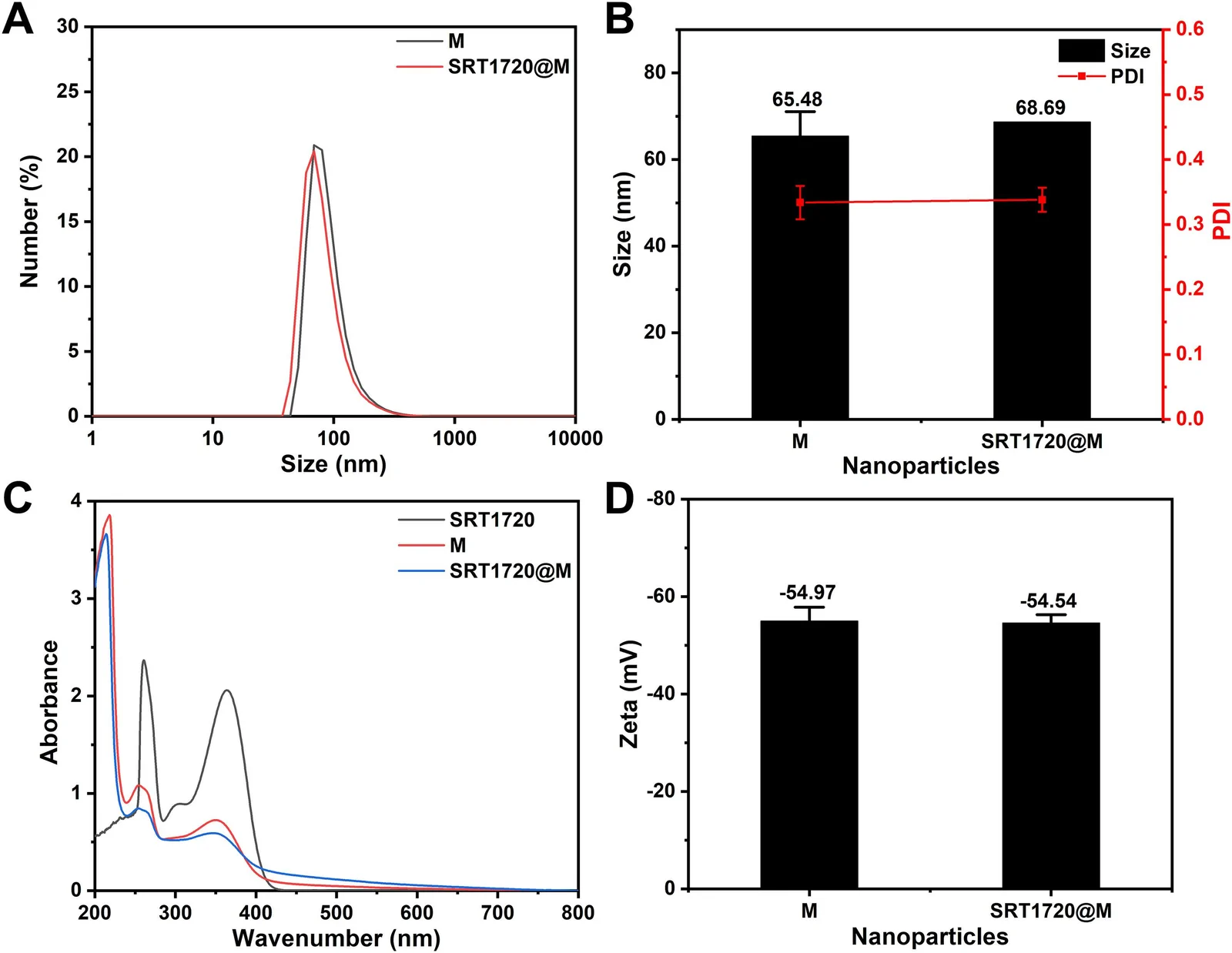
Synthesis and characterization of M and SRT1720@M nanovesicles. (A) Particle size distribution curves for M and SRT1720@M. (B) Average particle size and polydispersity index (PDI) values. (C) UV–Vis absorption spectra showing characteristic peaks of SRT1720 at 265 nm and 364 nm, retained in SRT1720@M. (D) Zeta potential measurements.

**Fig. 2:**
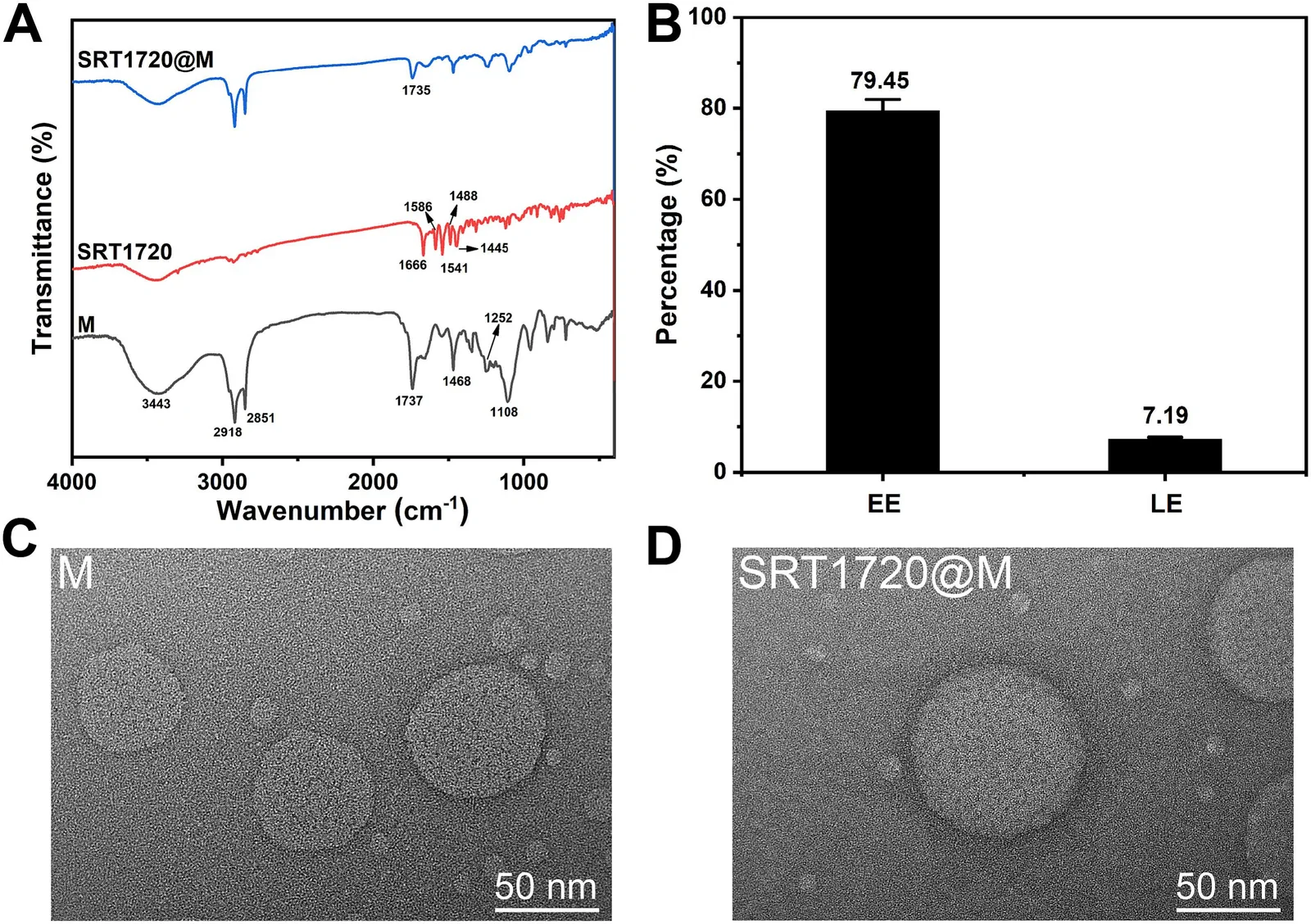
Further characterization of M and SRT1720@M. (A) FTIR spectra highlighting functional groups and interactions in M and SRT1720@M. (B) Encapsulation efficiency (EE) and loading efficiency (LE) of SRT1720@M. (C-D) Transmission electron microscopy (TEM) images of M (C) and SRT1720@M (D).

We successfully fabricated SRT1720@M as exosome-mimetic nanovesicles for brain-targeted delivery, optimizing their physicochemical properties to ensure systemic stability and improved blood-brain barrier (BBB) crossing. Dynamic light scattering (DLS) showed that the size of M and SRT1720@M were uniformly distributed between 50 and 500 nm, with most of them having size under 100 nm (Fig. 1A). In addition, loading with SRT1720 slightly increased the mean particle size from 65.48 nm (empty M) to 68.69 ± 3.21 nm for SRT1720@M (Fig. 1B). The size of these particles (between 50 and 100 nm) indicated the suitability for maximizing BBB penetration while reducing rapid clearance by the reticuloendothelial system. The polydispersity index (PDI) values (0.3338 for M and 0.3381 for SRT1720@M) were low (Fig. 1B), confirming a high level of structural regularity. UV–Vis spectra revealed that the signature peaks of SRT1720 at 265 nm and 364 nm were maintained in the SRT1720@M formulation without shifts (Fig. 1C), confirming that the drug's molecular structure remained undisturbed. Additionally, the zeta potential of SRT1720@M measured at −54.54 ± 2.13 mV (Fig. 1D), confirming robust electrostatic repulsion and colloidal stability, suggesting a low tendency to flocculate in in vivo-like fluids. The highly negative zeta potential (−54.54 ± 2.13 mV) confers several advantages: it reduces serum protein adsorption and subsequent reticuloendothelial system (RES) clearance via electrostatic repulsion, thereby prolonging systemic circulation time. Moreover, the negatively charged surface facilitates electrostatic interactions with positively charged sites on brain microvascular endothelial cells, promoting receptor-mediated transcytosis and enhancing blood-brain barrier (BBB) penetration. Moreover, FTIR spectra confirmed the phospholipid composition of the vesicle (Fig. 2A). At the same time, SRT1720@M displayed additional aromatic ring vibrations (1605–1445 cm^−1^) from the drug, suggesting robust lipid-drug integration and hydrogen bonding interactions that enhance cargo stability (Fig. 2A). Quantitative analysis using a validated standard curve showed an EE of 79.45 ± 2.10% and an LE of 7.19 ± 0.50% (Fig. 2B). TEM imaging showed that SRT1720@M nanoparticles were spherical, uniform particles (∼68 nm) without aggregation (Fig. 2C–D). These findings demonstrate that SRT1720@M is a stable, high-capacity biomimetic platform suitable for targeted delivery to the ischemic brain. The polydispersity index (PDI) of M and SRT1720@M was 0.3338 and 0.3381, respectively (Fig. 1B), indicating homogeneous size distribution. To assess colloidal stability, the nanovesicles were incubated in PBS (pH 7.4) and 10% FBS for 48 h; the particle size changed by less than 10%, and the PDI remained below 0.35 (Fig. S8A–B). The drug release profile of SRT1720@M showed cumulative releases of 62% at pH 7.4 and 78% at pH 5.5 over 48 h, demonstrating acid-accelerated release kinetics (Fig. S8C). Western blot analysis was performed to verify the exosome-mimetic identity of the nanovesicles. As shown in Fig. S14A–B, both M and SRT1720@M positively expressed the exosome markers CD63, CD81, TSG101, and Alix, whereas the endoplasmic reticulum marker Calnexin was negative. These results confirm that the prepared nanovesicles retain key characteristics of natural exosomes.

### 3.2 SRT1720@M enhances DCI biocompatibility and cell proliferation in OGD/R + HG model

**Fig. 3:**
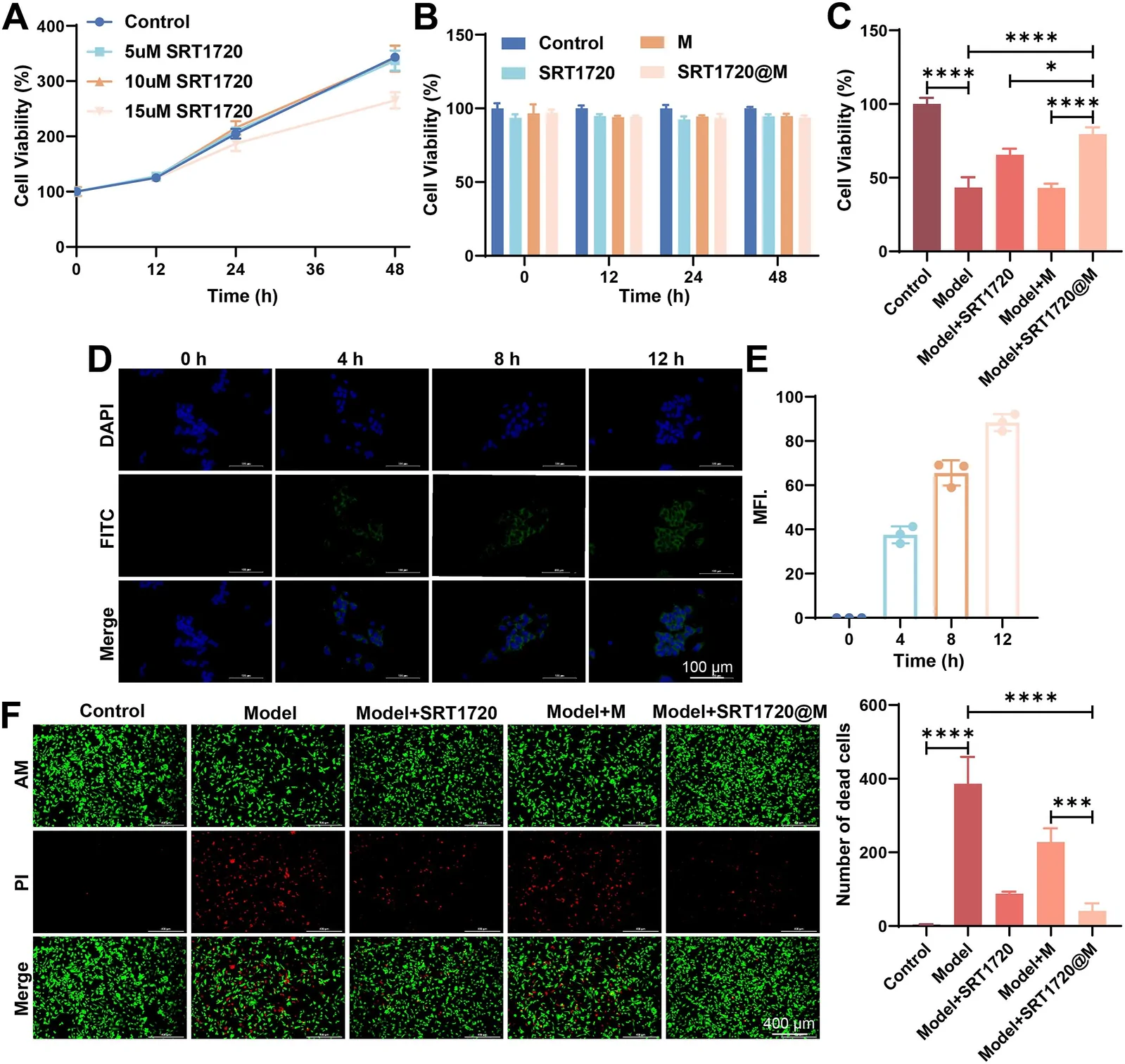
SRT1720@M promotes cell proliferation in primary hippocampal neurons. (A) CCK-8 assay screening for the optimal SRT1720 concentration (5–15 μM) after 24 h of treatment. (B) Cytotoxicity assessment of M and SRT1720@M over 0–48 h. (C) Cell viability in the OGD/R + HG model with treatments. (D-E) Cellular uptake of FITC-labeled SRT1720@M at 4, 8, and 12 h, shown by fluorescence images (D) and quantified mean fluorescence intensity (E). (F) Live/dead staining and ratio in control, model, and treatment groups. Data are mean ± SD (*n* = 3). *P < 0.05, ***P < 0.001, ****P < 0.0001 vs. control or model as indicated.

Primary hippocampal neurons were subjected to OGD/R + HG to assess the therapeutic potential of SRT1720@M. This two-insult model has been confirmed to have a high capacity to reproduce the hyperglycemia-ischemia synergy observed in DCI. The optimal dose of SRT1720 identified in the initial CCK-8 concentration screening was 10 μM; cell viability was unaffected by SRT1720 at doses between 5 and 10 μM after incubation for 24 h, but was significantly reduced at 15 μM (82.4 ± 4.1%, *p* < 0.05) (Fig. 3A). Biocompatibility assays also revealed no cytotoxicity with the empty M or SRT1720@M even after 48 h, and neuron viability remained over 95% at all hourly intervals (*P* > 0.05) relative to controls (Fig. 3B). CCK-8 assay also indicated that neuron viability dropped drastically in the OGD/R + HG group (*P* < 0.001 as compared to control), suggesting extreme cell damage. Free SRT1720 treatment counteracted the effect of OGD/R + HG, and this effect was further significantly promoted by SRT1720@M (*P* < 0.001 compared to model, *P* < 0.05 compared to free SRT1720, Fig. 3C), indicating enhanced internalization of the nanovesicles. As a matter of fact, cellular uptake of FITC-labeled SRT1720@M was found to increase over time (P < 0.001); Fig. 3D-E). These results were validated by live/dead staining, which revealed that there was a significant reduction in dead cells in SRT1720@M group as compared to model group (live/dead ratio: 88/12 vs. 60/40; *P* < 0.001; Fig. 3F). Overall, the findings suggest that neurons internalize SRT1720@M more effectively than free SRT1720 and is a better neuroprotectant against OGD/R + HG-inflicted damage.

Internalization pathway of SRT1720@M. To determine the cellular uptake mechanism, primary hippocampal neurons were pretreated with various endocytosis inhibitors before incubation with FITC-labeled SRT1720@M. Chlorpromazine (10 μg/mL) inhibits clathrin-mediated endocytosis, genistein (200 μM) inhibits caveolae-mediated uptake, and amiloride (1–2 mM) inhibits macropinocytosis. As shown in Fig. S15A–B, chlorpromazine and amiloride significantly reduced the intracellular fluorescence intensity of FITC-SRT1720@M (*P* < 0.01 compared with control), whereas genistein showed no significant effect. The DMSO solvent control did not affect uptake, and low temperature (4 °C) drastically diminished uptake. These results indicate that SRT1720@M is primarily internalized via clathrin-mediated endocytosis and macropinocytosis **(**Fig. S15).

### 3.3 SRT1720@M inhibits inflammation and activates SIRT1/PARKIN to promote mitophagy in vitro

**Fig. 4:**
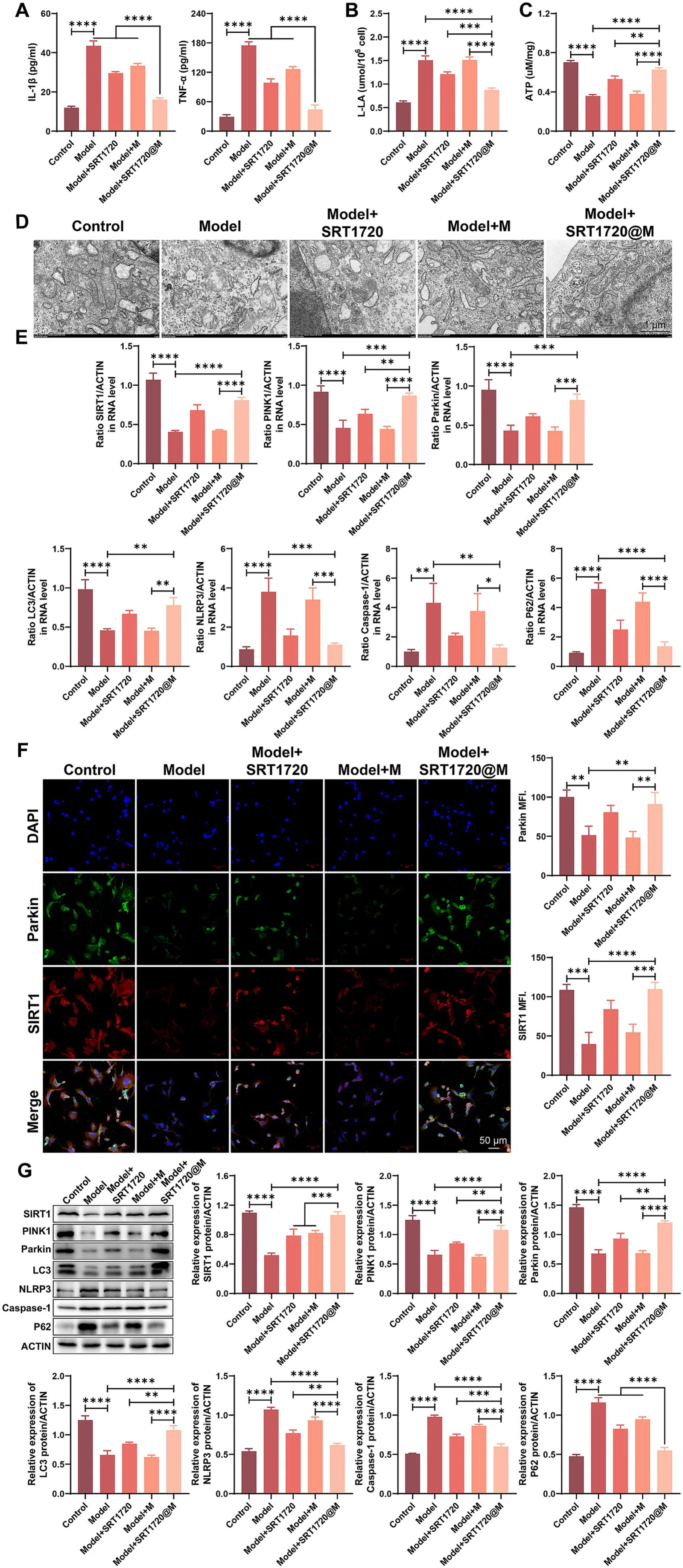
SRT1720@M activates the SIRT1/PARKIN pathway to promote mitophagy in the OGD/R + HG model in vitro. (A) ELISA detection of levels of IL-1β and TNF-α. (B) Determination of L-LA levels. (C) Determination of ATP levels. (D) TEM images of mitochondrial morphology in neurons. (E) qPCR expression of NLRP3, Caspase-1, SIRT1, PINK1, Parkin, LC3, and P62. (F) Immunofluorescence staining and quantification of SIRT1 and Parkin. (G) Western blot bands and densitometry for NLRP3, Caspase-1, SIRT1, PINK1, Parkin, LC3-I/II, and P62 (normalized to GAPDH). Data are mean ± SD (n = 3). *P < 0.05, **P < 0.01, ***P < 0.001, ****P < 0.0001 vs. control or model as indicated. Note: OGD/R + HG = oxygen-glucose deprivation/reperfusion + high glucose; M = blank exosome-mimetic nanovesicles; SRT1720@M = SRT1720-loaded exosome-mimetic nanovesicles; LC3-I/II = microtubule-associated protein 1 light chain 3-I/II; GAPDH = glyceraldehyde-3-phosphate dehydrogenase. Statistical analysis was performed using one-way ANOVA followed by Tukey's post-hoc test. Error bars represent standard deviation (SD). *P < 0.05, **P < 0.01, ***P < 0.001, ****P < 0.0001 indicate significant differences compared to the Control or Model group; #P < 0.05 indicates a significant difference between the Model+SRT1720@M and Model+SRT1720 groups.

The ELISA assay showed that OGD/R + HG induced significant inflammation, with increased IL-1 and TNF-α levels (*P* < 0.001 vs. control). SRT1720 treatment reduced these levels (P < 0.01). This effect was further enhanced by SRT1720@M (P < 0.001 vs. model, P < 0.05 vs. SRT1720) (Fig. 4A). Concerning energy metabolism, the L-LA level was upregulated (P < 0.001, Fig. 4B). In contrast, the ATP level was depleted (*P* < 0.001, Fig. 4C) in the model group. Notably, this effect was abrogated by SRT1720 and subsequently re-equilibrated by SRT1720@M (P < 0.01), confirming rehabilitation of mitochondrial activity (Fig. 4B and C). Mitochondrial swelling, loss of cristae, and rupture were observed in the model group, as revealed by TEM analysis (Fig. 4D). These changes were counteracted by SRT1720@M, in which mitochondrial diameter was reduced (*P* < 0.001) and cristae were intact in the majority of mitochondria (Fig. 4D), indicating improved mitochondrial clearance. Moreover, qPCR analysis showed that mitophagy markers (SIRT1, PINK1, PARKIN, and LC3) were downregulated while NLRP3, Caspase-1, and p62 were upregulated in the OGD/R + HG model but SRT1720@M reversed these effects; the effect of SRT1720@M was further promoted by SRT1720@M (Fig. 4E). Immunofluorescence analysis revealed that SIRT1 and PARKIN were downregulated in the model group (P < 0.001). Still, this effect was significantly reversed by SRT1720@M and SRT1720@M, and was notably pronounced with SRT1720@M@ (Fig. 4F, P < 0.001). Western blot results reflected the above expression changes in SIRT1, PINK1, PARKIN, LC3, NLRP3, Caspase-1, and P62 (Fig. 4G). These findings indicate that SRT1720@M mitigates mitochondrial injury by promoting SIRT1/PARKIN-mediated mitophagy.

To further confirm that SRT1720@M promotes mitophagy and neuroprotection specifically through the SIRT1/PARKIN pathway, we performed inhibition experiments using the selective SIRT1 inhibitor EX-527 (10 μM) or PARKIN siRNA (50 nM) in the OGD/R + HG model. As shown in Fig. S9A–B, treatment with EX-527 or si-PARKIN significantly attenuated the SRT1720@M-induced increase in the LC3-II/I ratio and cell viability, and reversed the decrease in p62 levels (*P* < 0.001 for all comparisons), restoring these parameters to levels comparable to the model group. These results confirm that the neuroprotective and mitophagy-enhancing effects of SRT1720@M are mediated via the SIRT1/PARKIN axis.

To further evaluate mitochondrial function, we measured mitochondrial ROS levels using mitoSOX staining and assessed mitochondrial membrane potential using JC-1 staining. As shown in Fig. S10A, OGD/R + HG treatment significantly increased mitoSOX fluorescence intensity (P < 0.001 vs. control), indicating elevated mitochondrial ROS production. Treatment with SRT1720@M markedly reduced this increase (P < 0.001 vs. model). JC-1 staining (Fig. S10B) revealed that OGD/R + HG caused a pronounced decrease in the red/green fluorescence ratio, indicating loss of mitochondrial membrane potential. SRT1720@M treatment restored the red/green ratio to a level comparable to the control group (P < 0.001 vs. model). These results confirm that SRT1720@M preserves mitochondrial integrity and function.

In addition to cytokine and inflammasome assessment, we examined NF-κB signaling. As shown in Fig. S11A–B, OGD/R + HG treatment increased the phosphorylation of NF-κB p65, indicating pathway activation. SRT1720@M treatment markedly reduced p-NF-κB p65 protein levels, further supporting its anti-inflammatory action.

To confirm that SRT1720@M promotes complete mitophagic flux rather than blocking autophagosome degradation, we performed a flux assay using bafilomycin A1 (baf A1, 100 nM, 4 h), which inhibits lysosomal acidification. As shown in Fig. S17A–B, treatment with baf A1 further increased LC3-II levels in the SRT1720@M group (*P* < 0.01), indicating enhanced autophagic flux. Additionally, p62 levels, which were decreased by SRT1720@M alone, significantly accumulated upon baf A1 treatment (P < 0.01), confirming that the autophagic degradation pathway is functionally intact. In contrast, the model group showed no significant change in p62 after baf A1, demonstrating blocked autophagic flux. These results unequivocally demonstrate that SRT1720@M promotes complete mitophagic flux (Fig. S17A-B).

To further confirm SIRT1 activation at the functional level, we measured SIRT1 deacetylase activity using a fluorometric assay. As shown in Fig. S18, SIRT1 enzymatic activity was significantly reduced in the OGD/R + HG group compared with control (P < 0.001). Treatment with SRT1720@M markedly restored SIRT1 activity (P < 0.001 vs. model), which was superior to free SRT1720 (*P* < 0.05). This effect was completely abolished by co-treatment with the SIRT1 inhibitor EX-527, confirming target specificity (Fig. S18).

### 3.4 Establishment and validation of the rat DCI model

**Fig. S1:**
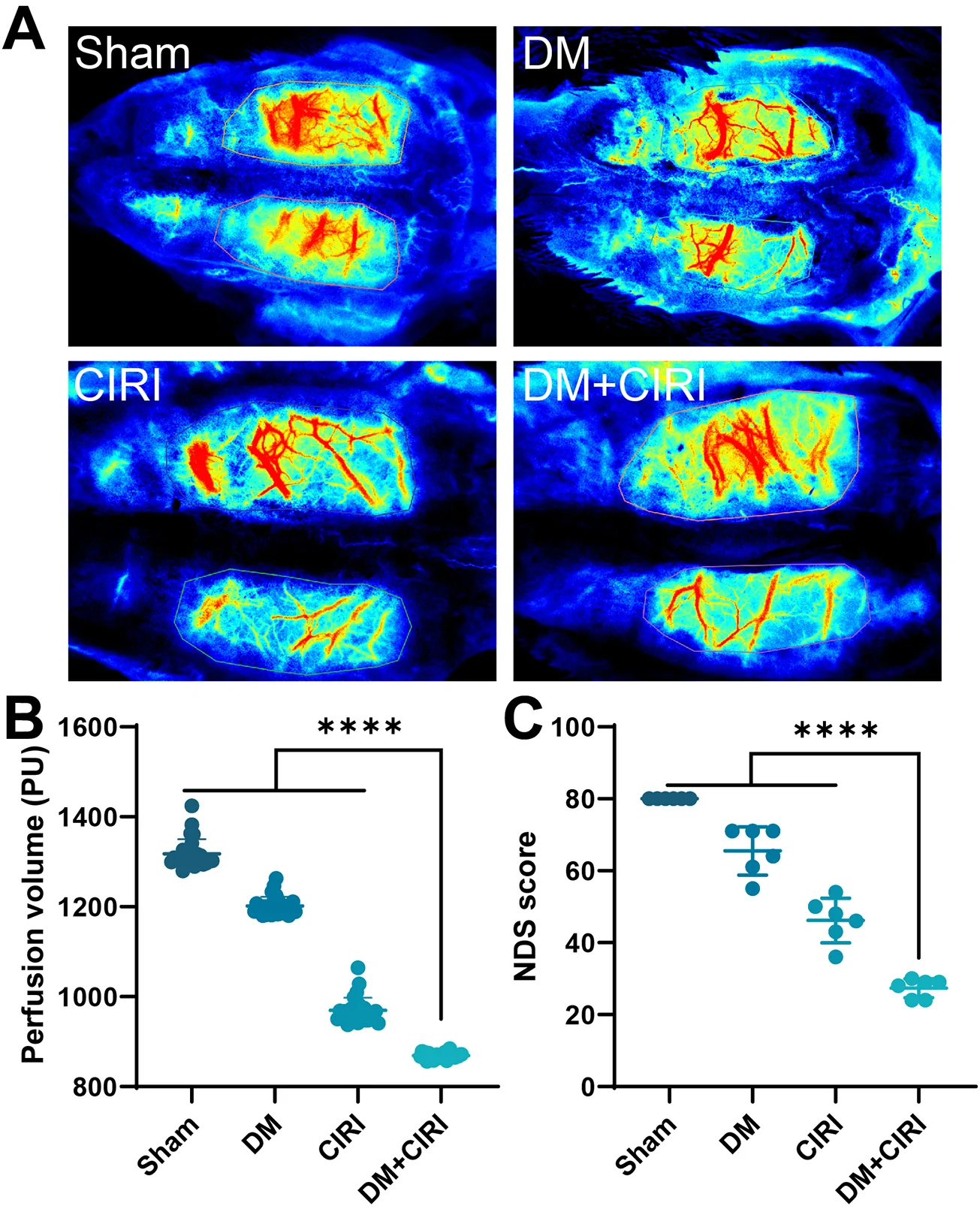
Validation of DCI model severity. (A-B) Laser Doppler flowmetry monitoring of rat brain blood flow imaging (A) and quantitative perfusion analysis (B) in Sham, DM, CIRI, and DM + CIRI groups. (C) NDS scoring. *n* = 6, *P < 0.05, **P < 0.01, ***P < 0.001, ****P < 0.0001 vs. Sham.

**Fig. S2:**
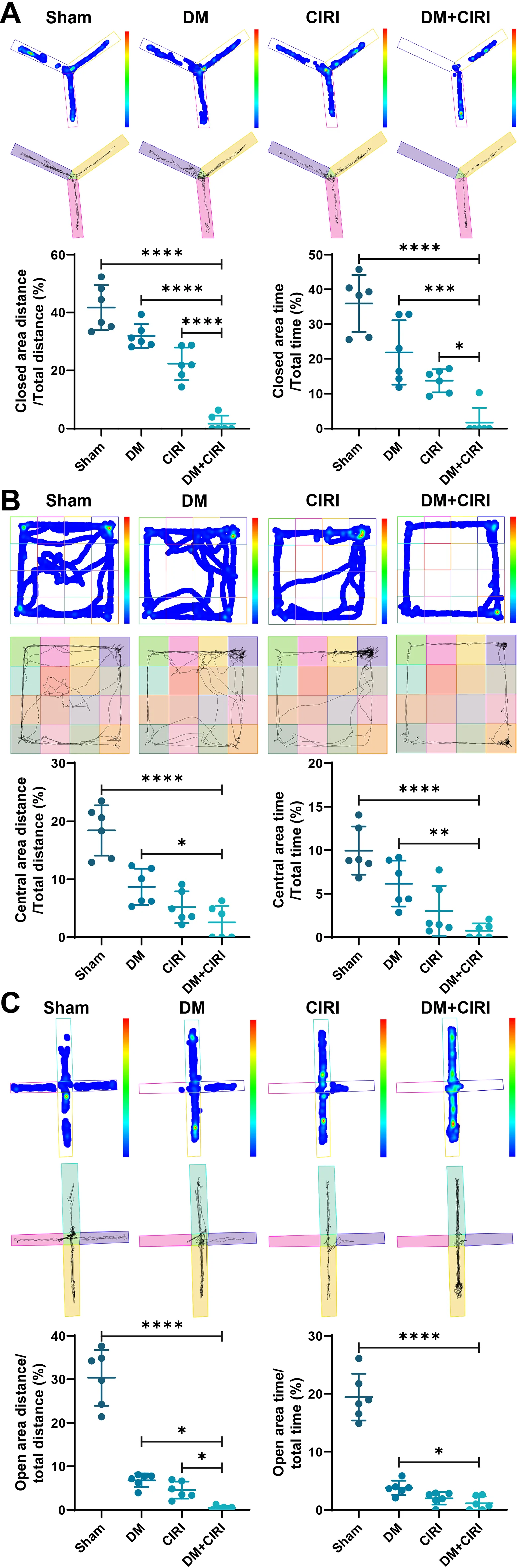
Behavioral impairments in the DCI model. (A-C) Y-maze novel arm exploration, open field central zone activity, and elevated plus maze open arm entries in Sham, DM, CIRI, and DM + CIRI groups. *n* = 6, *P < 0.05, **P < 0.01, ***P < 0.001, ****P < 0.0001 vs. Sham.

**Fig. S3:**
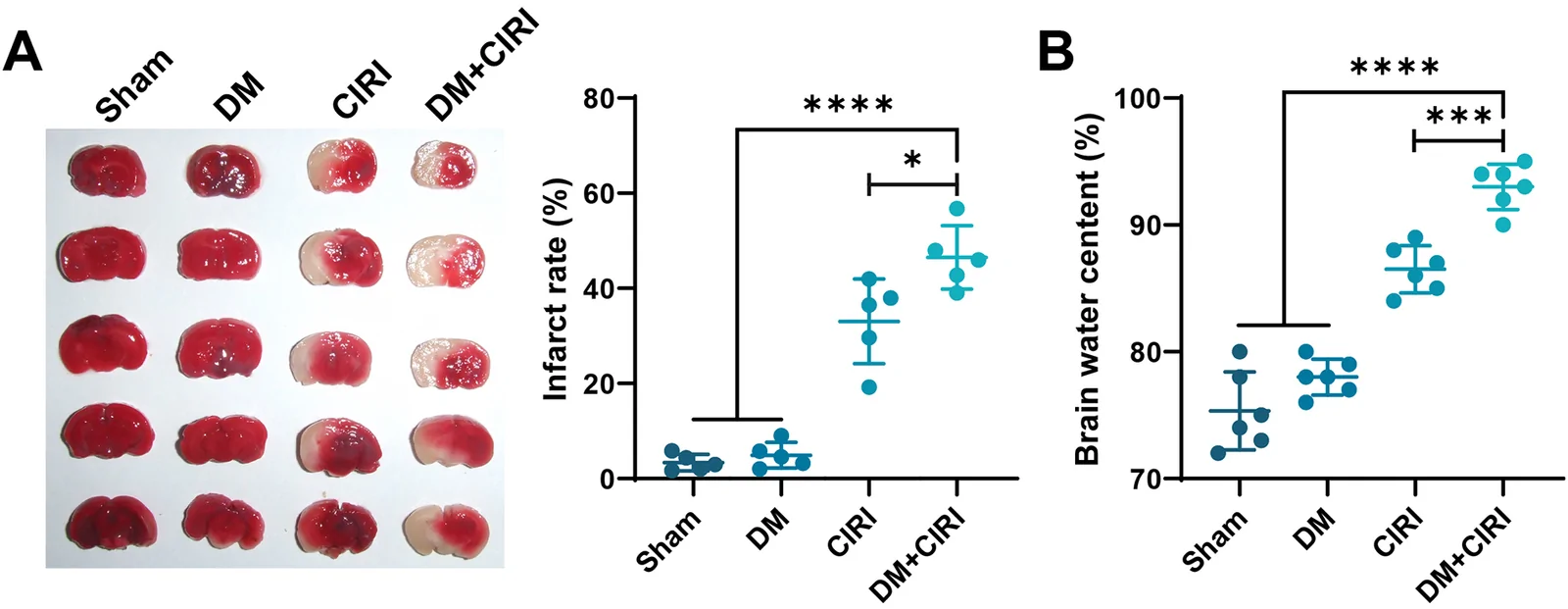
Infarct and edema in the DCI model. (A) TTC staining for infarct volume. (B) Brain water content. n = 6, *P < 0.05, **P < 0.01, ***P < 0.001, ****P < 0.0001 vs. Sham.

**Fig. S4:**
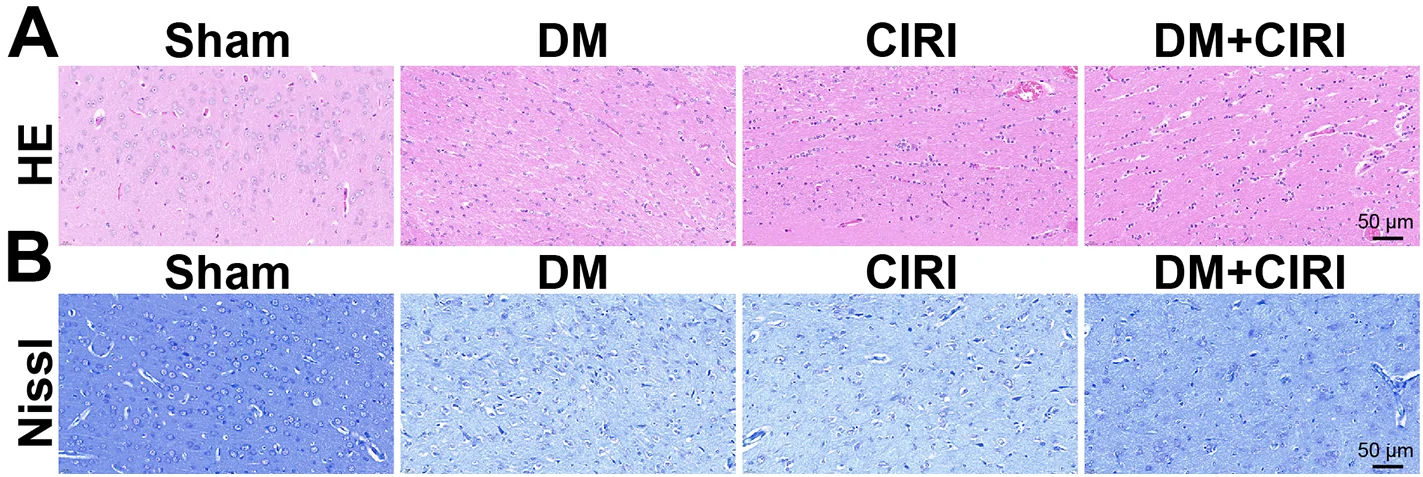
Histopathological changes in the DCI model. (A) H&E staining showing neuronal arrangement and morphology. (B) Nissl staining for Nissl bodies. *n* = 3, *P < 0.05, **P < 0.01, ***P < 0.001, ****P < 0.0001 vs. Sham.

**Fig. S5:**
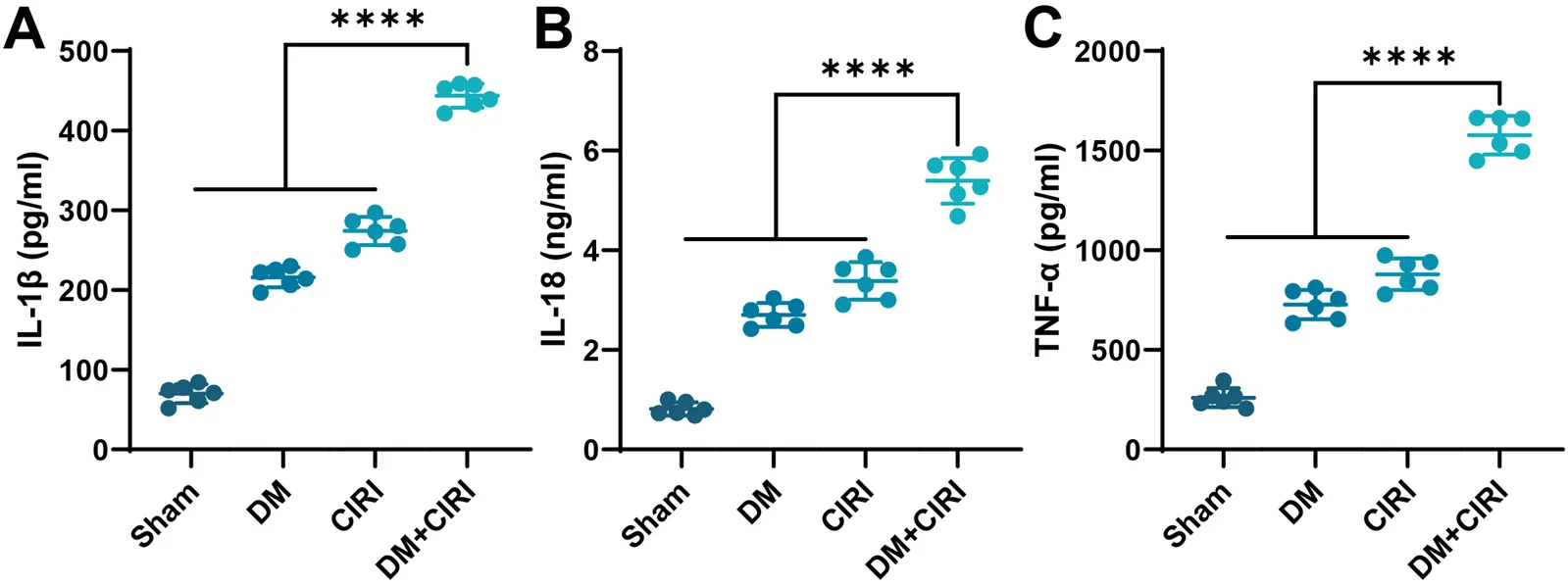
Inflammatory markers in the DCI model. ELISA detection of (A) IL-1β, (B) IL-18, and (C) TNF-α expression levels in Sham, DM, CIRI, and DM + CIRI groups. n = 6, *P < 0.05, **P < 0.01, ***P < 0.001, ****P < 0.0001 vs. Sham.

**Fig. S6:**
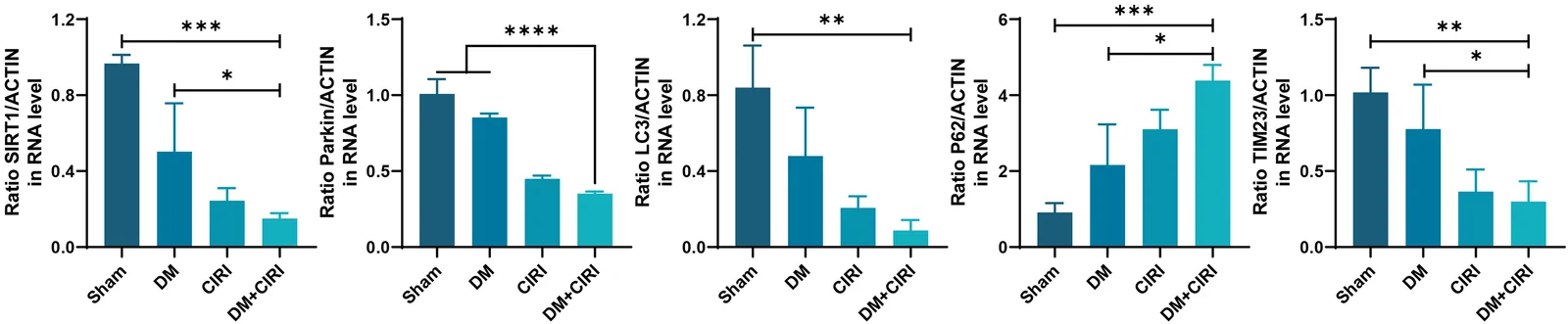
Gene expression changes in the mitophagy pathway. qPCR detection of SIRT1, Parkin, LC3, P62, and TIM23 gene expression in Sham, DM, CIRI, and DM + CIRI groups. *n* = 3, *P < 0.05, **P < 0.01, ***P < 0.001, ****P < 0.0001 vs. Sham.

**Fig. S7:**
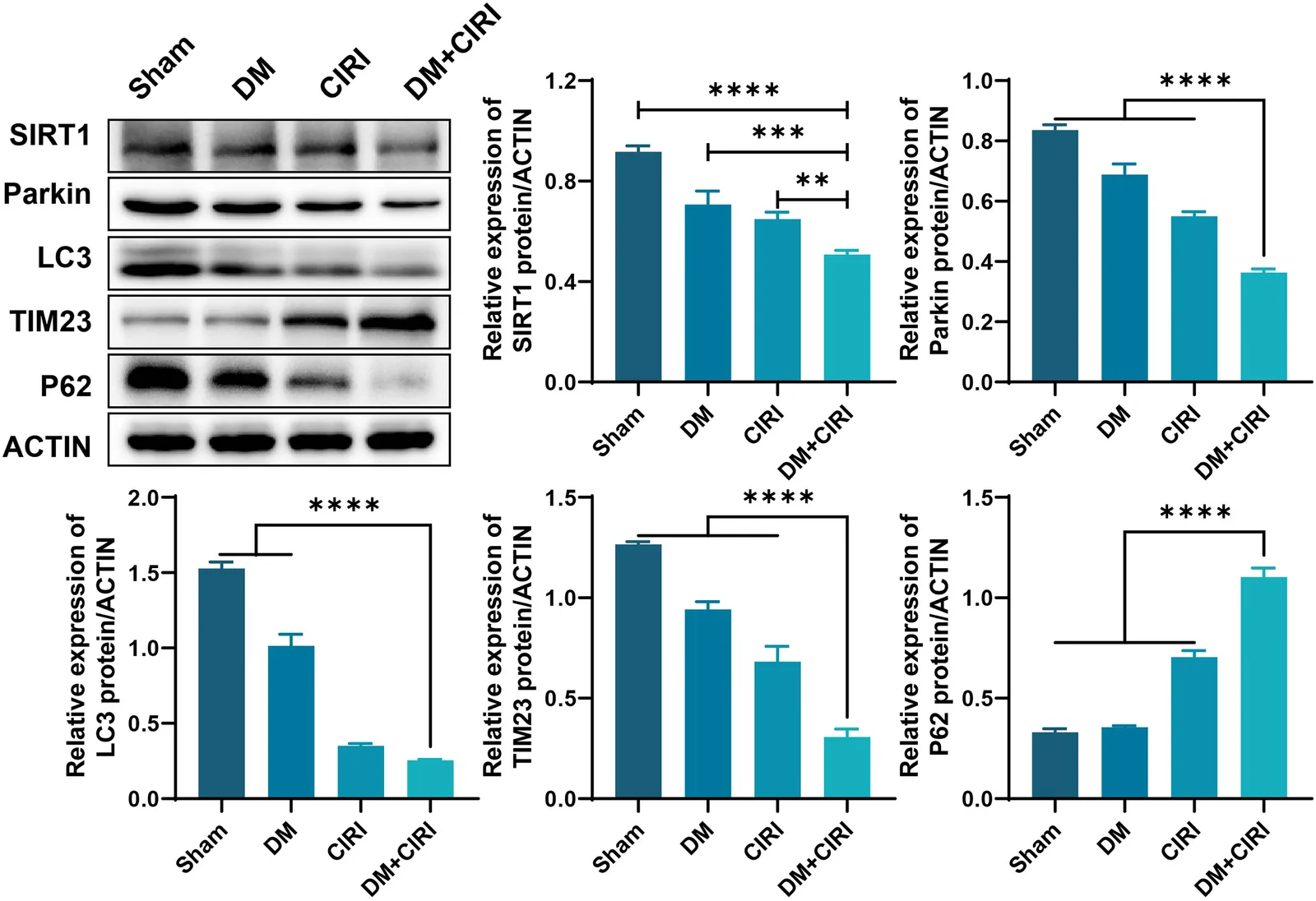
Protein expression changes in the mitophagy pathway. Western blot detection of SIRT1, Parkin, LC3, P62, and TIM23 protein expression in Sham, DM, CIRI, and DM + CIRI groups. n = 3, *P < 0.05, **P < 0.01, ***P < 0.001, ****P < 0.0001 vs. Sham.

For in vivo experimental verification, we established and validated the DCI model by assessing biologically relevant behavioral and molecular parameters. Laser Doppler flowmetry was leveraged to monitor cortical perfusion. The results showed that although DM and CIRI alone reduced blood flow, the DM + CIRI group exhibited the most pronounced deficit in cerebral perfusion (Fig. S1**A**). Quantitative analysis showed a significant reduction in blood flow in the DCI group compared with sham (*p* < 0.0001; Fig. S1**B**). Correspondingly, the NDS was highest in the DM + CIRI group, suggesting severe motor and sensory impairment attributable to the comorbid condition (Fig. S1**C**). Cognitive and exploratory deficits were assessed through a battery of behavioral tests. In the y-maze, the DM + CIRI rats showed significantly less interest in the novel arm, suggesting impaired spatial cognition (Fig. S2A). Furthermore, open field tests revealed depleted central zone homeostasis (Fig. S2B), and elevated plus maze trials showed fewer open arm entries (Fig. S2C), collectively indicating exacerbated stress-related behavior and reduced exploratory drive in the DCI model (*p* < 0.0001). Macro-structural insult-induced atrophy was quantified by means of TTC staining, which highlighted significantly widened infarct volumes in the DM + CIRI group compared to either DM or CIRI alone (Fig. S3A). This structural insult-induced atrophy was accompanied by a critical peak in brain water content, confirming that the DCI model causes severe cerebral fluid accumulation (Fig. S3B). Histological examinations were employed to verify cellular-level evidence of injury. H&E staining in the DM + CIRI group showed architecturally compromised neuronal arrangement, pyknotic nuclei, and vacuolization (Fig. S4A). Nissl staining subsequently substantiated these findings, showing a marked depletion of Nissl bodies and a significant reduction in neuronal number in the DCI model compared with the highly organized sham group (Fig. S4B). The inflammatory response was characterized by measuring key pro-inflammatory cytokines via ELISA. the levels of IL-1β, IL-18, and TNF-α were all significantly elevated in the DM + CIRI group (Fig. S5A–C). Notably, the combined insult of diabetes and ischemia-reperfusion triggered a compounded inflammatory spike that was significantly higher than that observed in the CIRI-only group (*p* < 0.001). Finally, we used qPCR to assess transcriptional homeostasis of the mitophagy pathway. in the DM + CIRI group, the expression of pro-mitophagy genes SIRT1, PARKIN, LC3, and TIM23 was significantly downregulated, while the autophagy substrate p62 was upregulated (Fig. S6). These results indicate acute impairment of mitochondrial quality control and autophagic homeostatic throughput in the DCI brain, identifying the SIRT1/PARKIN axis as a primary therapeutic target. Western blot analysis was implemented to establish whether the observed transcriptional changes were reflected and maintained at the protein level (Fig. S7). Collectively, these data (Fig. S1, Fig. S2, Fig. S3, Fig. S4, Fig. S5, Fig. S6, Fig. S7) validate that the combination of diabetes and MCAO successfully recapitulates the exacerbated injury profile of DCI, distinguishing it from standard ischemic stroke. Congruent with established qPCR findings, protein expression of SIRT1, PARKIN, LC3, and the mitochondrial marker TIM23 was significantly reduced in the DM + CIRI group compared with the sham group (p < 0.0001). Furthermore, p62 protein sequestration was notably higher in the DCI model (*p* < 0.001), a definitive marker of impaired autophagic homeostatic throughput. These protein-level results reiterate that the combined diabetic and ischemic insult induce a robust disruption of the SIRT1/PARKIN-mediated mitophagy axis, thereby validating the molecular baseline of the DCI rat brain.

### 3.5 SRT1720@M protects against neurological and mitochondrial injury in DCI rats

**Fig. 5:**
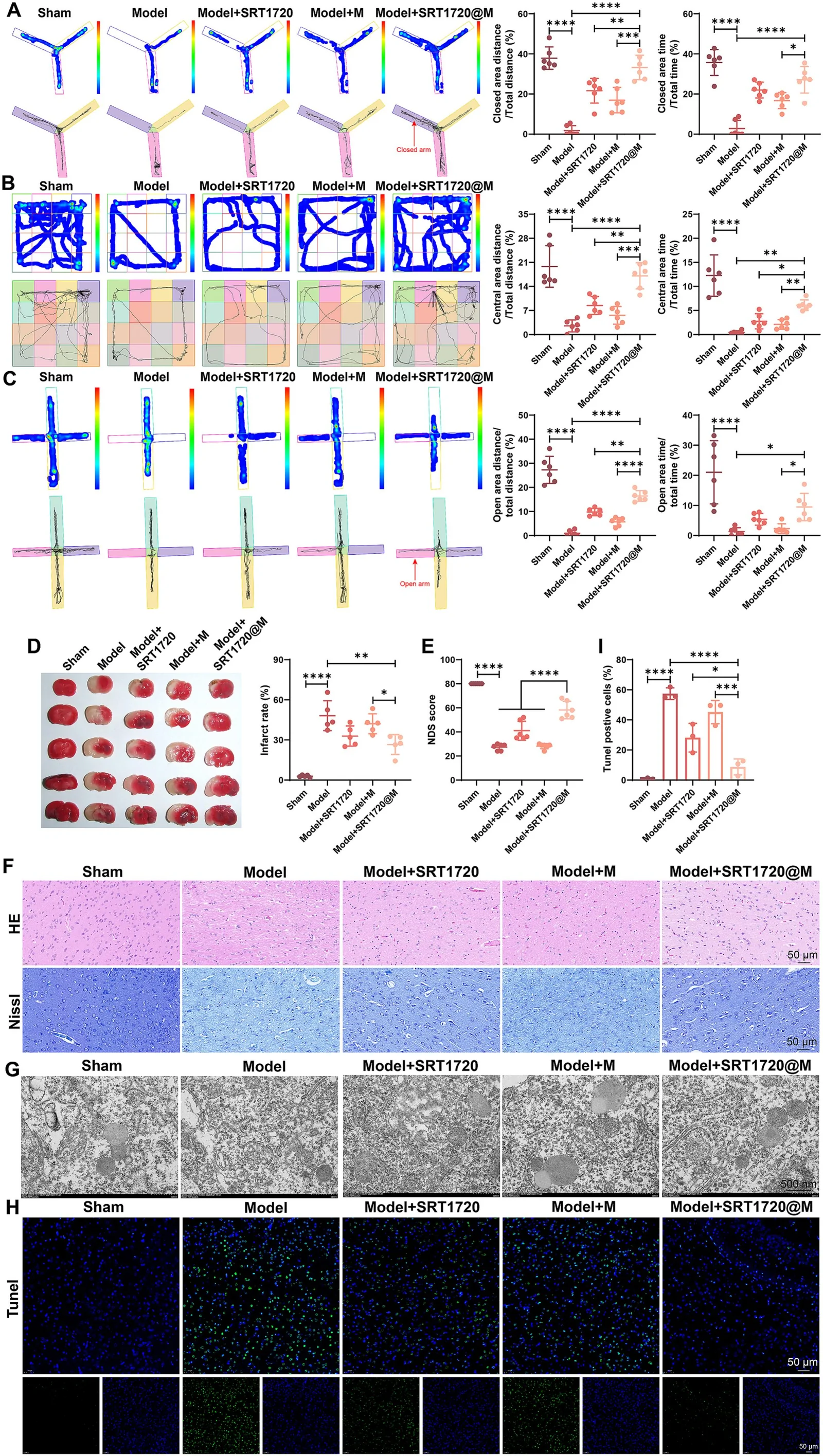
SRT1720@M rescues behavioral, neurological, and mitochondrial damage in DCI rats. (A) Y-maze test showing representative movement trajectory maps from each group and corresponding percentage of entries and percentage of cumulative time spent in the novel arm. (B) Open field test showing representative movement trajectory maps, the percentage of distance moved within the central zone, and the percentage of time spent exploring within the central zone. (C) Elevated plus maze (EPM) test showing paths of animals in EPM, open arm entries, and open arm time. (D) TTC staining and infarct volume quantification. (E) NDS scores. (F) H&E staining of brain tissue. (G) TEM images of brain mitochondrial morphology. (H-I) TUNEL staining images (H) and apoptotic index quantification. (I) Data are mean ± SD (*n* = 6 for behavior/NDS/TTC/TUNEL; n = 3 for TEM). *P < 0.05, **P < 0.01, ***P < 0.001, ****P < 0.0001 in compared groups.

To assess the potential benefits of SRT1720@M, we evaluated functional and structural improvements in DCI rats across multiple biological scales. DCI significantly impaired spatial memory and exploratory behavior, effects that SRT1720 and SRT1720@M reversed. In the Y-maze assessment (Fig. 5A), DCI and empty M-treated rats showed a reduced preference for the novel arm (p < 0.001); nevertheless, SRT1720 and, more markedly, SRT1720@M, increased both the percentage of entries and the cumulative time spent in the novel arm, suggesting an improvement of short-term spatial recognition memory. Furthermore, in the OFT (Fig. 5B), DCI rats showed increased preference for the perimeter, whereas SRT1720 and, more markedly, SRT1720@M displayed greater distance and time spent exploring the central zone, suggesting decreased anxiety; this anxiolytic action was further validated in the EPM (Fig. 5C, p < 0.001), where SRT1720 and, more markedly, SRT1720@M increased the frequency of open-arm entries and total open-arm time compared with the DCI group (p < 0.001).

Structural brain damage was measured to evaluate the neuroprotective effect of SRT1720@M. TTC staining (Fig. 5D) displayed increased cerebral infarction in the DCI and DCI + M groups, whereas both SRT1720 and SRT1720@M markedly decreased the total infarct volume (p < 0.001). These results correlated with neurological outcomes, as evidenced by a marked decrease in NDS in both SRT1720- and SRT1720@M-treated rats (Fig. 5E; p < 0.001). H&E staining (Fig. 5F) showed that both SRT1720 and SRT1720@M treatments normalized neuronal density and tissue architecture, thereby alleviating vacuolation and pyknosis detected in the DCI group. In addition, TEM results (Fig. 5G) showed that DCI induced mitochondrial swelling, loss of mitochondrial crests, and membrane rupture. By contrast, both SRT1720 and SRT1720@M effectively preserved mitochondrial morphology, maintaining more intact cristae and double membranes. TUNEL staining results (Fig. 5H) and corresponding quantification of the TUNEL-positive cells (Fig. 5I) demonstrated a marked rise in apoptosis rate in the DCI group (p < 0.001). Both SRT1720 (p < 0.001) and SRT1720@M (p < 0.001) markedly inhibited apoptosis, with SRT1720@M producing the most significant reduction (p < 0.001 compared with SRT1720). These findings emphasize the neuroprotective effectiveness of SRT1720@M, which surpasses that of free SRT1720. The enhanced benefits of SRT1720@M, likely due to its sustained release and efficient BBB penetration, demonstrate its potential to improve neurological function and safeguard brain tissue in the DCI rat model. These in vivo results show that SRT1720@M effectively crosses the blood-brain barrier, offers superior neuroprotection compared to free SRT1720, and enhances neurological function and brain tissue integrity in DCI rats.

### 3.6 SRT1720@M protects against neurological and mitochondrial injury in DCI rats

**Fig. 6:**
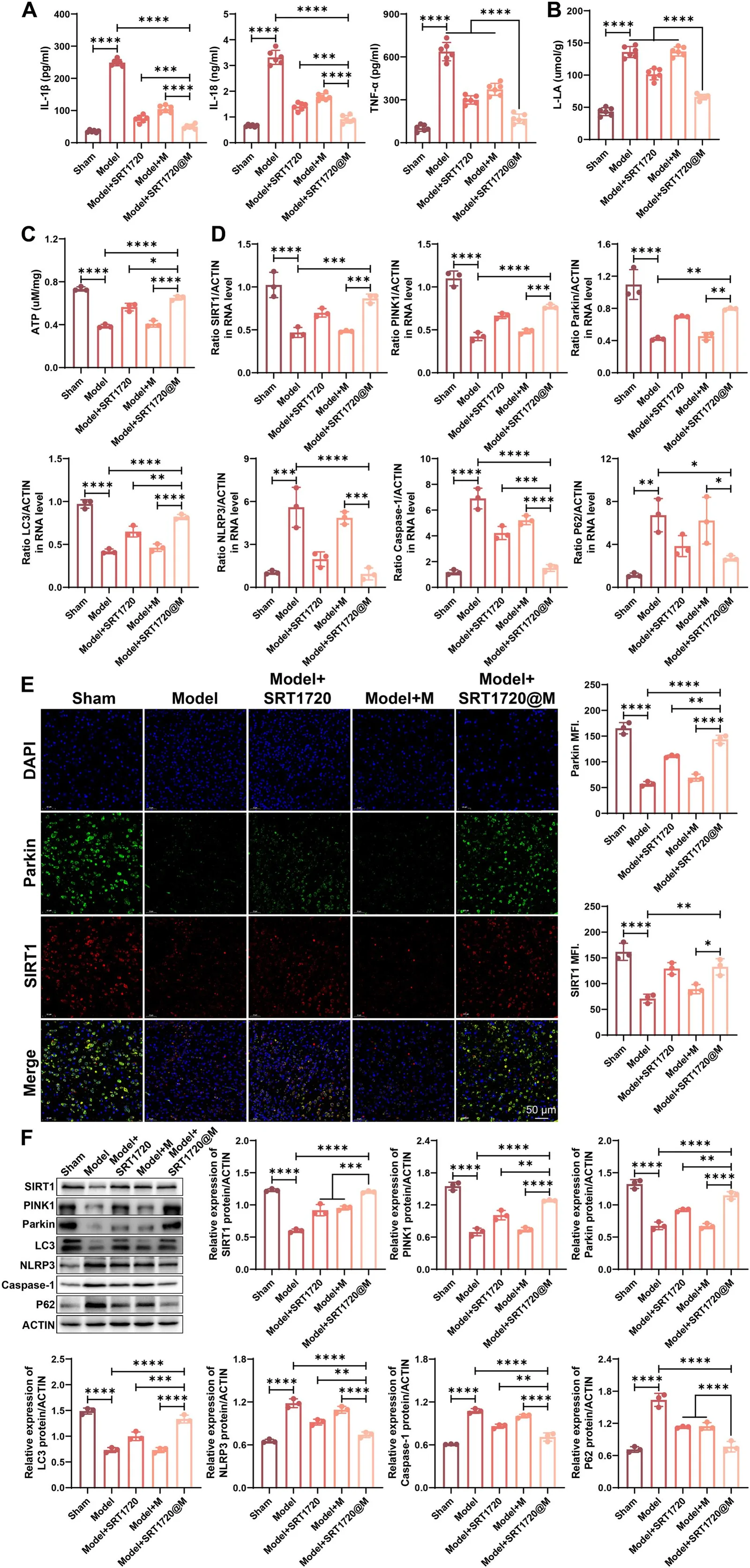
SRT1720@M activates the SIRT1/PARKIN pathway to promote mitophagy in the DCI rat brain. (A) ELISA levels of IL-1β, IL-18, and TNF-α. (B) L-LA levels. (C) ATP levels. (D) qPCR expression of NLRP3, Caspase-1, SIRT1, PINK1, Parkin, LC3, and P62. (E) Immunofluorescence staining and quantification of SIRT1 and Parkin. (F) Western blot bands and densitometry for NLRP3, Caspase-1, SIRT1, PINK1, Parkin, LC3-I/II, and P62 (normalized to β-actin). Data are mean ± SD (n = 3). *P < 0.05, **P < 0.01, ***P < 0.001, ****P < 0.0001 vs. Sham or Model as indicated.

In vivo biocompatibility and safety assessment. To evaluate the systemic safety of SRT1720@M, we performed histological and serum biochemical analyses. As shown in Fig. S13A, H&E staining of major organs (heart, liver, spleen, lung, and kidney) revealed no obvious pathological abnormalities in the SRT1720@M group compared with the sham group. Furthermore, serum levels of alanine aminotransferase (ALT), aspartate aminotransferase (AST), blood urea nitrogen (BUN), and creatinine (Cr) were not significantly different between the SRT1720@M group and control groups (Fig. S13B). Additionally, serum pro-inflammatory cytokines IL-6 and TNF-α were not elevated in SRT1720@M-treated animals (Fig. 6A), indicating no acute inflammatory response. These findings collectively demonstrate the favorable in vivo safety profile of SRT1720@M.

### 3.7 SRT1720@M activates SIRT1/PARKIN signaling-mediated mitophagy and alleviates neuroinflammation in rats

To elucidate the molecular mechanisms underlying the neuroprotective effects of SRT1720@M, we evaluated mitochondrial homeostasis and inflammatory signaling in the DCI rat brain (Fig. 6). ELISA analysis revealed that DCI induced a marked increase in pro-inflammatory cytokine levels, including IL-1β, IL-18, and TNF-α. While SRT1720 partially attenuated these effects, the SRT1720@M group exhibited the most significant reduction in the levels of these cytokines (*p* < 0.0001), suggesting a synergistic anti-inflammatory effect (Fig. 6A). We also assessed the metabolic state in the brain by measuring L-LA (Fig. 6B) and ATP (Fig. 6C) levels. DCI caused a sharp decline in L-LA (Fig. 6B) and ATP (Fig. 6C), indicating damaged aerobic metabolism. Treatment with SRT1720@M successfully rescued these metabolic parameters, significantly increasing ATP (Fig. 6C, *p* < 0.01) relative to the model, a critical cofactor for SIRT1 activity. The qPCR analysis showed that DCI triggered the upregulation of NLRP3, Caspase-1, as well as the mitochondrial fission marker PINK1 (Fig. 6D, p < 0.01). Simultaneously, mitophagy-related genes Parkin, LC3, and P62 were downregulated in the DCI model group (Fig. 6D, p < 0.01). Notably, the SRT1720@M treatment significantly suppressed NLRP3 and Caspase-1 expression while robustly upregulating Parkin, LC3, and P62 (Fig. 6D; p < 0.01). PINK1 expression was also notably increased, suggesting it may serve as a downstream mediator of SIRT1 (Fig. 6D, p < 0.01). To confirm activation of the mitophagy pathway, we performed immunofluorescence staining and Western blot analysis (Fig. 6E–F). Immunofluorescence showed that SIRT1 and PARKIN expression were severely diminished in the DCI model brain (Fig. 6E, *p* < 0.05). The SRT1720@M treatment significantly restored the fluorescent intensity and colocalization of SIRT1 and PARKIN, indicating enhanced recruitment of PARKIN to damaged mitochondria (Fig. 6E; p < 0.05). These findings were corroborated by densitometric analysis of Western blots (Fig. 6F; p < 0.05), which showed that SRT1720@M significantly increased protein levels of SIRT1, PARKIN, and autophagy markers (Parkin, LC3, and P62), whereas it decreased levels of NLRP3 and Caspase-1. Together, these results indicate that SRT1720@M effectively promotes SIRT1/PARKIN-mediated mitophagy, thereby reducing neuroinflammation and metabolic dysfunction in the DCI rat brain. These findings suggest that SRT1720@M activates the SIRT1/PARKIN-mediated mitophagy pathway, which is crucial for reducing DCI pathology. The improved targeting of nanovesicles to the brain likely explains the greater efficacy of SRT1720@M compared with free SRT1720, resulting in better penetration and sustained therapeutic effects. In conclusion, SRT1720@M can target the brain, induce SIRT1/PARKIN-mediated mitophagy in neurons, inhibit neuroinflammation and apoptosis, restore mitochondrial function, and ultimately alleviate DCI in vivo.

## 4 Discussion

DCI poses significant challenges in both clinical and socio-economic areas because it results from complex interactions between metabolism and vascular problems. Major hurdles include the blood-brain barrier, low drug bioavailability, and the progressive nature of the disease, underscoring the need for effective treatments. In the present study, we showed that SRT1720@M exosome-mimetic nanoparticle formulation displays excellent systemic stability, efficient brain targeting, and beneficial neuroprotective outcomes in DCI models. SRT1720@M markedly enhanced the viability of neuronal cells, attenuated neuroinflammation, repaired the function of mitochondria, and elicited SIRT1/PARKIN-regulated mitophagy in vitro and in vivo. Additionally, SRT1720@M provided greater restorative benefits than free SRT1720, as evidenced by improved behavioral outcomes, smaller infarct volumes, reduced neuronal apoptosis, and better mitochondrial health in DCI rats. These findings underscore the potency of SRT1720@M as a novel nano-based strategy for treating DCI by targeting mitochondrial quality control and neuroinflammatory pathways. By combining advanced nanotechnology with precise drug delivery, SRT1720@M presents a promising option for treating neurovascular issues in diabetic patients.

The BBB limits the delivery of small-molecule therapeutics, with about 98% unable to cross into neural tissue (Sharma et al., 2025). BBB maintains the structure and function of tight-junction proteins, which form a physical barrier within endothelial cells to protect against toxic metabolites and counteract neurotoxicity (Kuo et al., 2021) The constant stress on the cerebral microvasculature caused by chronic hypertension and the absence of nocturnal blood-pressure dipping make the BBB susceptible, increasing the risk of symptomatic intracerebral hemorrhage after ischemic injuries (Chen et al., 2025; Long et al., 2025). In addition, acute disruption of the BBB during infarction facilitates the release of synaptic proteins, such as PSD95. These factors, in addition to poor solubility, low bioavailability, and rapid clearance, limit the clinical application of SRT1720, a potent activator of SIRT1 that provides neuroprotection by enhancing mitochondrial function and decreasing oxidative stress (Jiang et al., 2023; Sun et al., 2025a; Xing et al., 2024). To overcome these physiological barriers, we encapsulated SRT1720 within exosome-mimetic nanoparticles. This method improved delivery to brain tissue, reduced pro-inflammatory cytokines, and regulated mitochondrial turnover. SRT1720@M exosome-mimetic formulation enhances this delivery method by leveraging the natural biocompatibility and barrier-crossing abilities of exosome-based structures. This improved delivery system enables sustained concentrations of the SIRT1 activator at the infarction site, overcoming the limitations of free small molecules. By mimicking the formation and surface characteristics of endogenous exosomes, these vesicles exhibit improved colloidal stability and a highly negative zeta potential (−54.54 mV), thereby reducing flocculation and enhancing systemic survival. Moreover, The encapsulation efficiency of SRT1720 in our exosome-mimetic formulation reached 79.45%, which is superior to that of most liposomal SRT1720 systems reported in the literature (typically ranging from 55% to 70%). This value is comparable to PLGA-based nanoparticles (approximately 75–82%), but our system offers the additional advantages of enhanced biocompatibility and superior blood-brain barrier penetration owing to its biomimetic design. These features collectively underscore the technological significance of SRT1720@M as a highly efficient brain-targeted delivery platform for diabetic cerebral infarction therapy (Wu et al., 2023).

These features improve colloidal stability, prevent particle aggregation, and increase systemic circulation time. By using endomembrane trafficking signals and mimicking the size of natural exosomes (30–150 nm), SRT1720@M crosses the BBB more effectively than other nanodelivery systems, such as mesoporous silica or basic polymeric micelles (Sun et al., 2025b). Unlike traditional small molecules, which are usually rapidly cleared by the liver, the SRT1720@M nanoparticles utilize receptor-mediated endocytosis, enabling them to cross the BBB and reach the CNS. This is particularly crucial for treating DCI in diabetic patients, as their BBB is frequently impaired due to chronic hypertension and vascular problems (Jiang et al., 2020; Li et al., 2020; Ning et al., 2017; Yuen et al., 2019; Zhang et al., 2016; Zhou et al., 2022). Furthermore, the ability of nanovesicles to stabilize tight junction proteins, such as occludin and ZO-1, supports the preservation of BBB integrity and reduces the risk of contrast-induced neurotoxicity, which frequently occurs during diagnostic imaging procedures (Jiang et al., 2020; Li et al., 2020; Simon Machado et al., 2023; Tang et al., 2022; Zhang et al., 2025; Zhou et al., 2022). Our findings also demonstrate that SRT1720@M enhances both cognitive and motor functions in animal models of DCI. Psychomotor assessments suggest it improves spatial memory and alleviates anxiety, which are essential markers of neurological impairment. Structural analyses showed that SRT1720@M markedly reduces infarct size, restores neuronal density, and enhances mitochondrial structure, all of which are vital for neuronal survival and recovery.

A critical feature of this study is the efficacy of SRT1720@M to stimulate selective mitophagy via the SIRT1/PARKIN axis. Previous studies highlight the role of mitophagy in mitochondrial homeostasis during oxidative stress (Hou et al., 2025; Hwang et al., 2024; Lan et al., 2024; Li et al., 2023; Liu et al., 2024a; Mao et al., 2022). In DCI, restoring mitophagic flux reduces mitochondrial dysfunction, a crucial factor in ischemic injury (Hou et al., 2025; Hwang et al., 2024; Lan et al., 2024; Li et al., 2023; Liu et al., 2024a; Mao et al., 2022). Our findings are consistent with other research showing that enhancing mitophagy can improve tissue repair and functional recovery in ischemic models. The ability of SRT1720@M to target the SIRT1/PARKIN cascade provides a new curative approach for treating mitochondrial dysfunction, which is a key feature of DCI in diabetic patients. Our study introduces a mitophagy-enhanced method that mimics the specificity of Mito-MEN technology, a system designed to direct PARKIN mRNA-complexed nanoparticles to mitochondria, thereby promoting the selective clearance of damaged organelles and simultaneously increasing exogenous delivery to mitochondria (Wang et al., 2024). Encapsulating SRT1720 in an exosome-like matrix can help solve the long-standing solubility and low bioavailability issues that have limited the clinical use of free resveratrol or synthetic SIRT1 activators. This nanocarrier offers targeted precision similar to PINK1 siRNA-loaded PLGA nanoparticles, which have been successfully used to treat neurological conditions, deliver specifically to microglia, reduce neuroinflammation and excessive mitophagy, and stabilize infarct volume to enhance functional outcomes (Xu et al., 2023). This integrated nanotherapy combines metabolic stabilization and neuroprotection to support neuronal survival during hyperglycemic-ischemic stress by promoting SIRT1/PARKIN mitochondrial turnover and inhibiting NLRP3 inflammasome activity, which often causes neuronal death in diabetics. The ability of SRT1720@M to target the SIRT1/PARKIN cascade provides a new curative approach for treating mitochondrial dysfunction, which is a key feature of DCI in diabetic patients. Activation of the SIRT1/PARKIN pathway, which restores mitochondrial bioenergetics and breaks the cycle of oxidative stress and ion imbalance.

The clinical benefits of SRT1720@M included modulating the immune response of the brain. It decreased NLRP3 inflammasome activation, inhibiting the inflammatory cascade that worsens tissue damage after cerebral ischemia. This anti-inflammatory effect is essential for decreasing reperfusion injury, a process that can cause more tissue damage than the initial ischemic episode. SRT1720@M promotes mitochondrial renewal, reduces oxidative damage, lowers the risk of reperfusion injury, and improves long-term DCI outcomes. Besides its neuroprotective benefits, the SRT1720@M formulation provides notable advantages in patient adherence and ease of access to treatment. Diabetic stroke patients, particularly from lower socioeconomic backgrounds, frequently face challenges such as limited healthcare access, inadequate nutrition, and low health literacy. These factors contribute to the progression of diabetic complications and delay the start of intervention. The cost-effective, highly efficient SRT1720@M nanoplatform can be a potential option, offering a practical intervention for high-risk groups. Additionally, the ability of SRT1720@M to restore the neurovascular system and enhance microvascular function makes it a promising candidate for addressing the metabolic and neurovascular issues observed in diabetic patients with DCI.

Study limitations. However, several limitations require further study. While we demonstrated short-term safety and efficacy (up to 7 days), long-term (>4 weeks) outcomes and stroke-healing durability remain unknown. Future studies should examine immune responses, off-target effects, and release kinetics of nanovesicle-delivered molecules to ensure long-term potency. Moreover, the scalability of exosome-mimetic nanovesicle production for GMP has not been validated, and BBB penetration requires confirmation in non-human primates. Addressing these limitations will advance clinical translation of this nanoplatform.

## 5 Conclusion

The SRT1720@M exosome-mimetic nanoparticle formulation advances treatment for DCI. By effectively restoring mitochondrial function, fostering mitophagy, and inhibiting neuroinflammation, SRT1720@M boosts neuronal survival and accelerates functional recovery. This study emphasizes the potential of exosome-based drug delivery systems as a promising method to overcome barriers posed by traditional therapies, such as the BBB. It provides a practical solution for enhancing outcomes in high-risk diabetic stroke patients. With its cost-effectiveness and high efficiency, SRT1720@M could help address the global impact of diabetic stroke and open new avenues.

The following are the supplementary data related to this article.

**Fig. S8:**
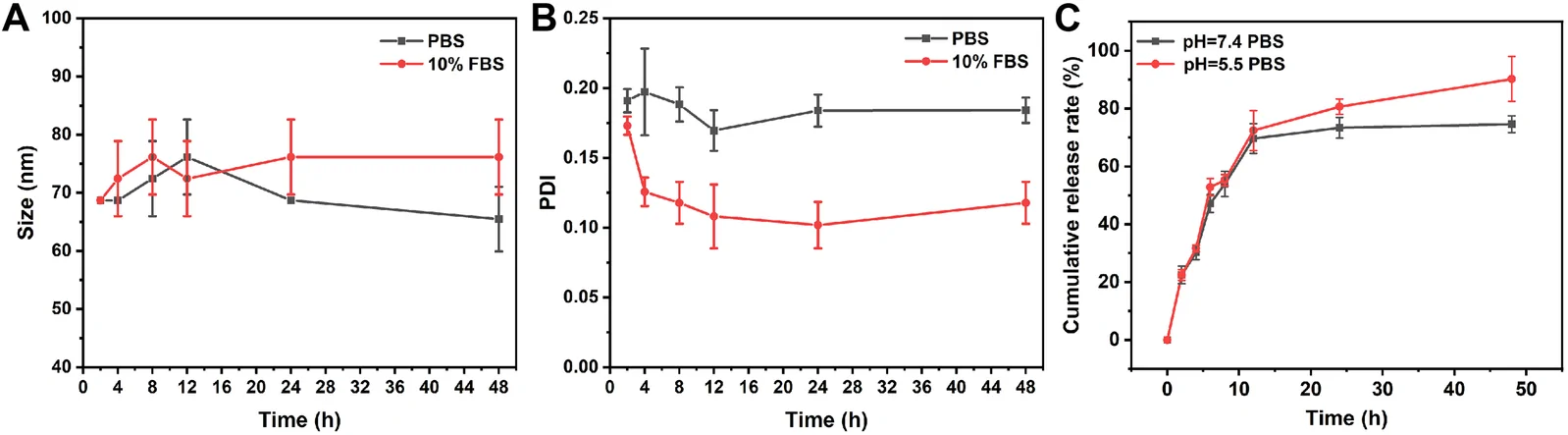
Protein expression changes in mitophagy pathway. Western blot detection of SIRT1, Parkin, LC3, P62, and TIM23 protein expression in Sham, DM, CIRI, and DM + CIRI groups. n = 3, *P < 0.05, **P < 0.01, ***P < 0.001, ****P < 0.0001 vs. Sham.

**Fig. S9:**
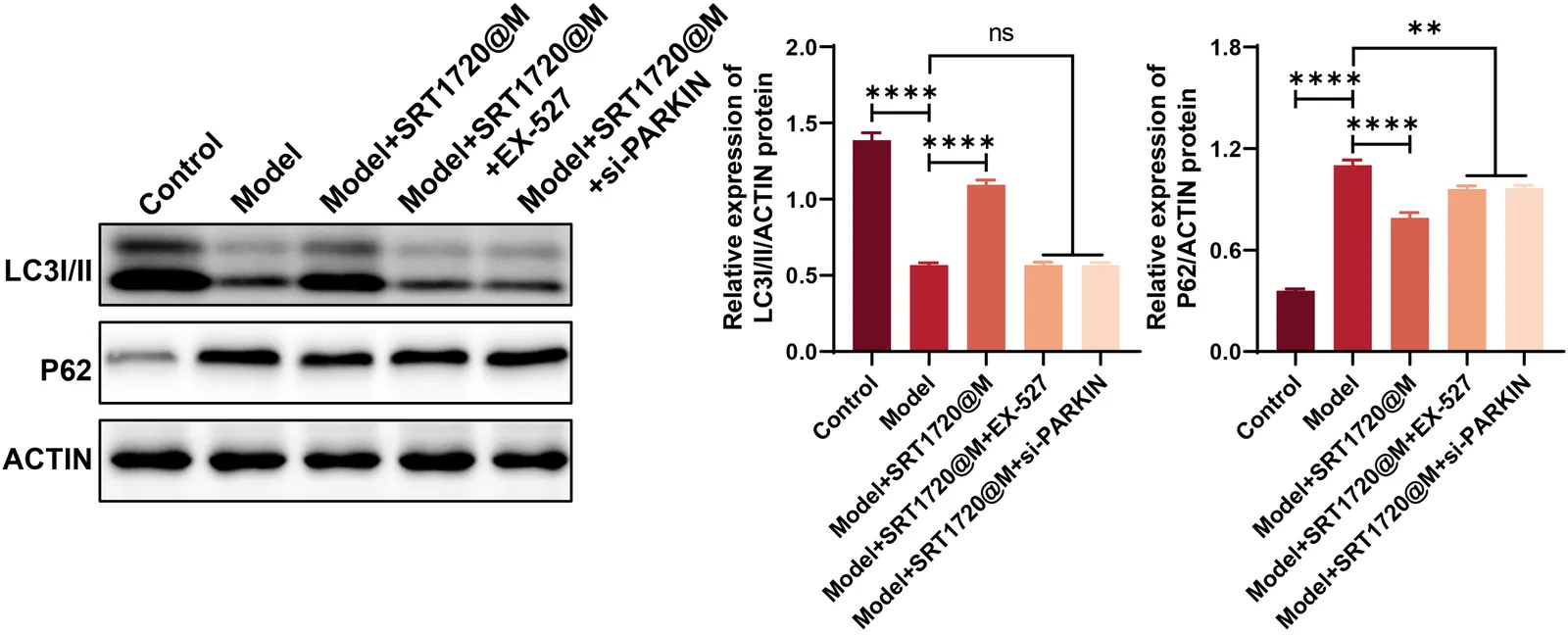
Inhibition of SIRT1 or PARKIN abolishes the neuroprotective and mitophagy-promoting effects of SRT1720@M in the OGD/R + HG model. (A) Representative Western blots of LC3-I/II and p62. (B) Quantification of LC3-II/I ratio, p62 levels, and cell viability (CCK-8). Groups: Control, Model (OGD/R + HG), Model+SRT1720@M, Model+SRT1720@M + EX-527 (10 μM), and Model+SRT1720@M + si-PARKIN (50 nM). Data are mean ± SD (n = 3). ***P < 0.001 vs. Control; Statistical analysis: one-way ANOVA with Tukey's post-hoc test.

**Fig. S10:**
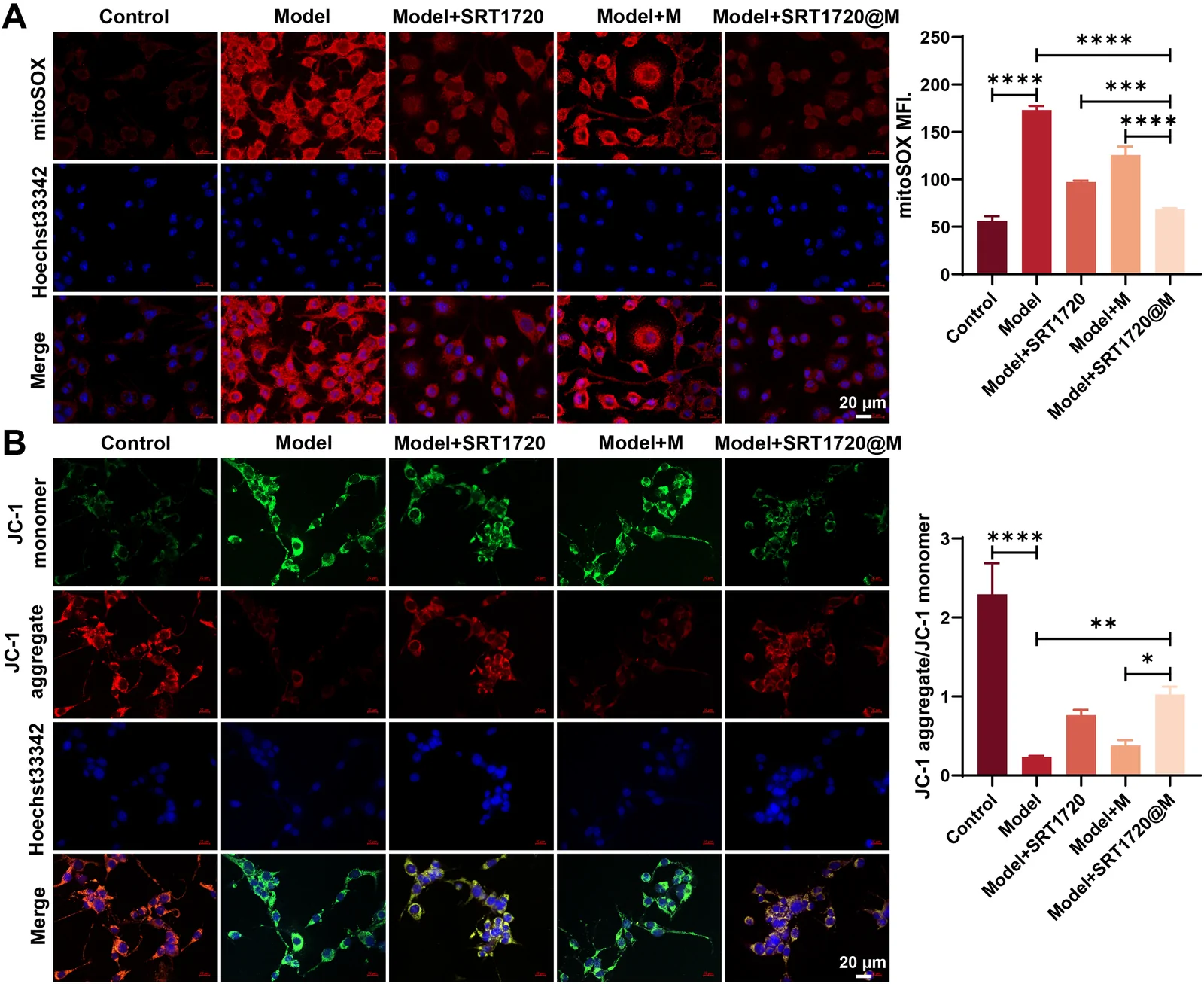
SRT1720@M reduces mitochondrial ROS and restores mitochondrial membrane potential in the OGD/R + HG model. (A) Representative mitoSOX fluorescence images and quantitative analysis of mitochondrial ROS levels. (B) Representative JC-1 staining images and quantification of red/green fluorescence ratio. Groups: Control, Model (OGD/R + HG), Model+SRT1720@M. Data are mean ± SD (n = 3). ***P < 0.001 vs. Control. Scale bars = 20 μm.

**Fig. S11:**
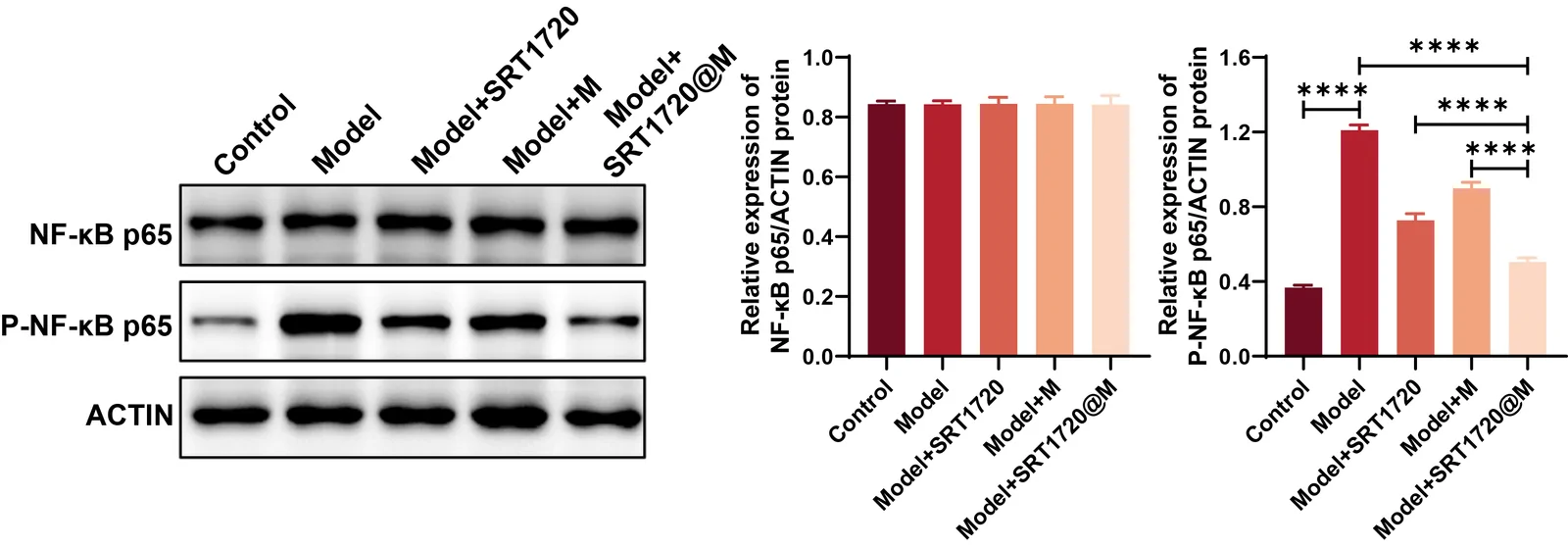
SRT1720@M inhibits NF-κB p65 phosphorylation in the OGD/R + HG model. (A) Representative Western blots of NF-κB p65 and p-NF-κB p65. (B) Quantitative analysis of p-NF-κB/NF-κB ratio. Groups: Control, Model (OGD/R + HG), Model+SRT1720, Model+M, Model+SRT1720@M. Data are mean ± SD (n = 3). *P < 0.05, **P < 0.01, ***P < 0.001 vs. Model.

**Fig. S12:**
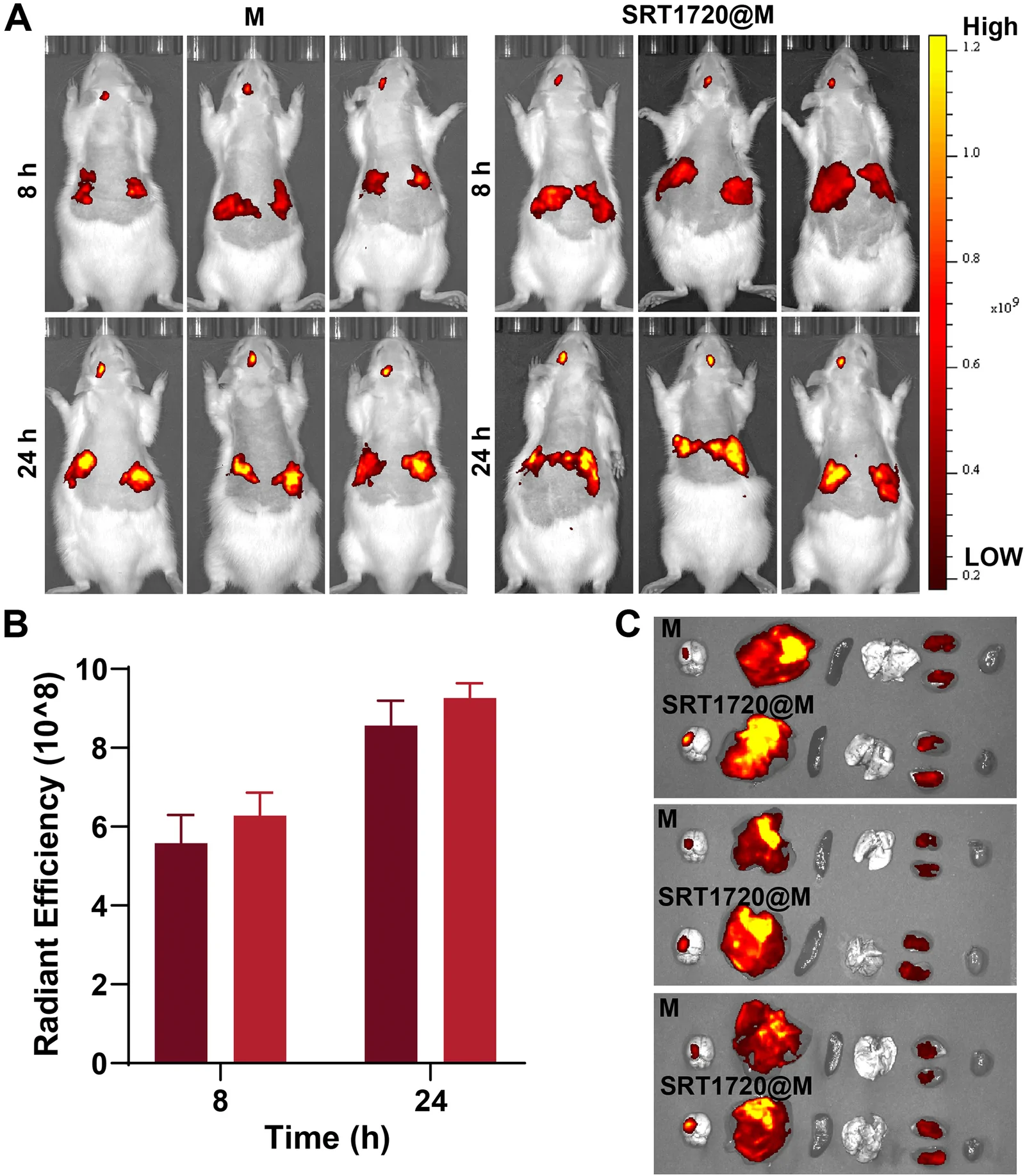
In vivo brain accumulation and BBB penetration of SRT1720@M. (A) Representative in vivo fluorescence images of DCI rats at 8 h and 24 h after intravenous injection of free DiR or DiR-labeled SRT1720@M. (B) Quantitative analysis of brain fluorescence intensity (n = 3). ***P < 0.001 vs. free DiR group.

**Fig. S13:**
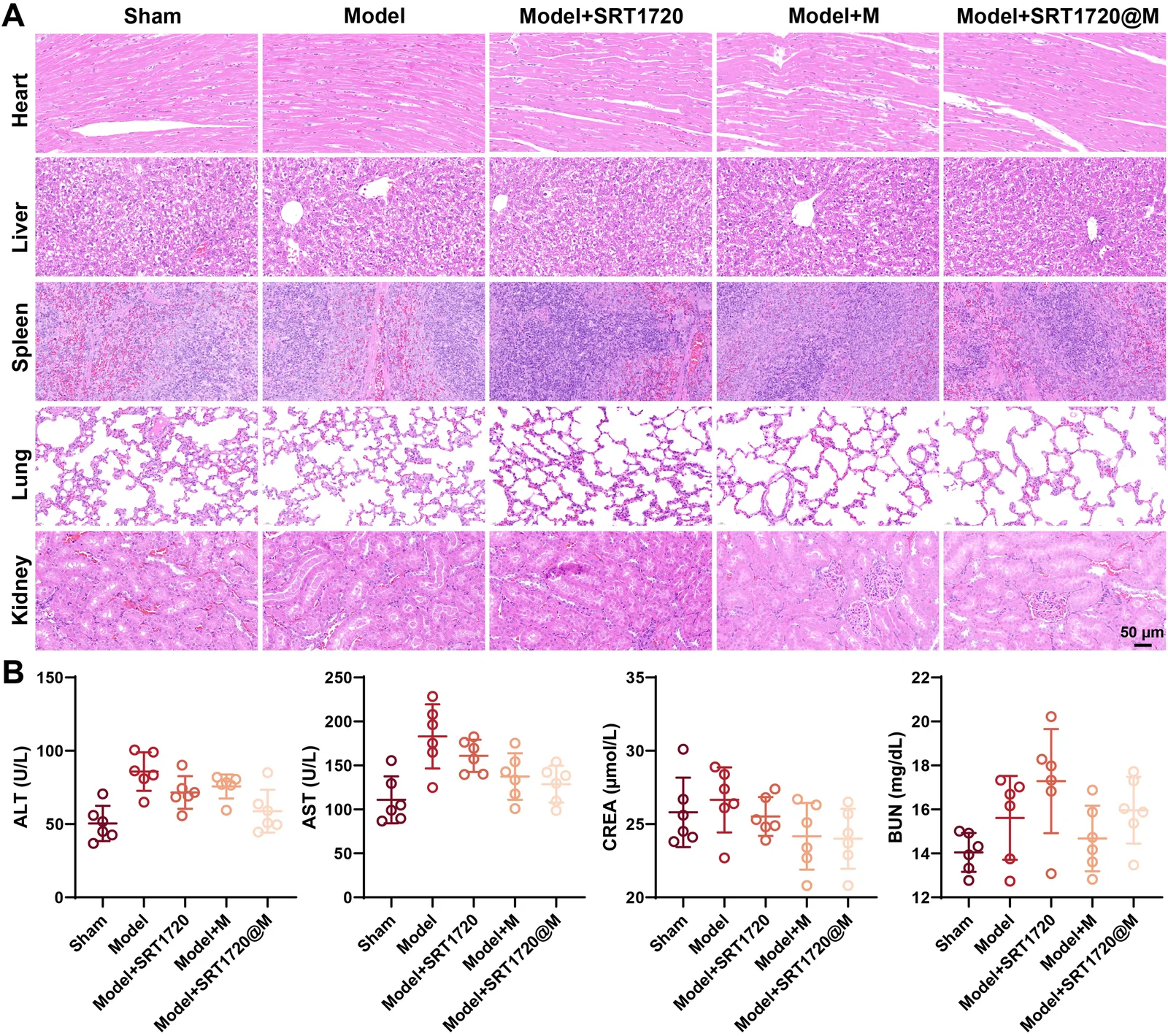
In vivo biocompatibility and safety of SRT1720@M. (A) Representative H&E staining images of heart, liver, spleen, lung, and kidney from each group (scale bar = 50 μm). (B) Serum biochemical parameters: alanine aminotransferase (ALT), aspartate aminotransferase (AST), blood urea nitrogen (BUN), and creatinine (Cr). Data are mean ± SD (n = 6 per group). n.s. indicates no significant difference compared with sham group (one-way ANOVA).

**Fig. S14:**
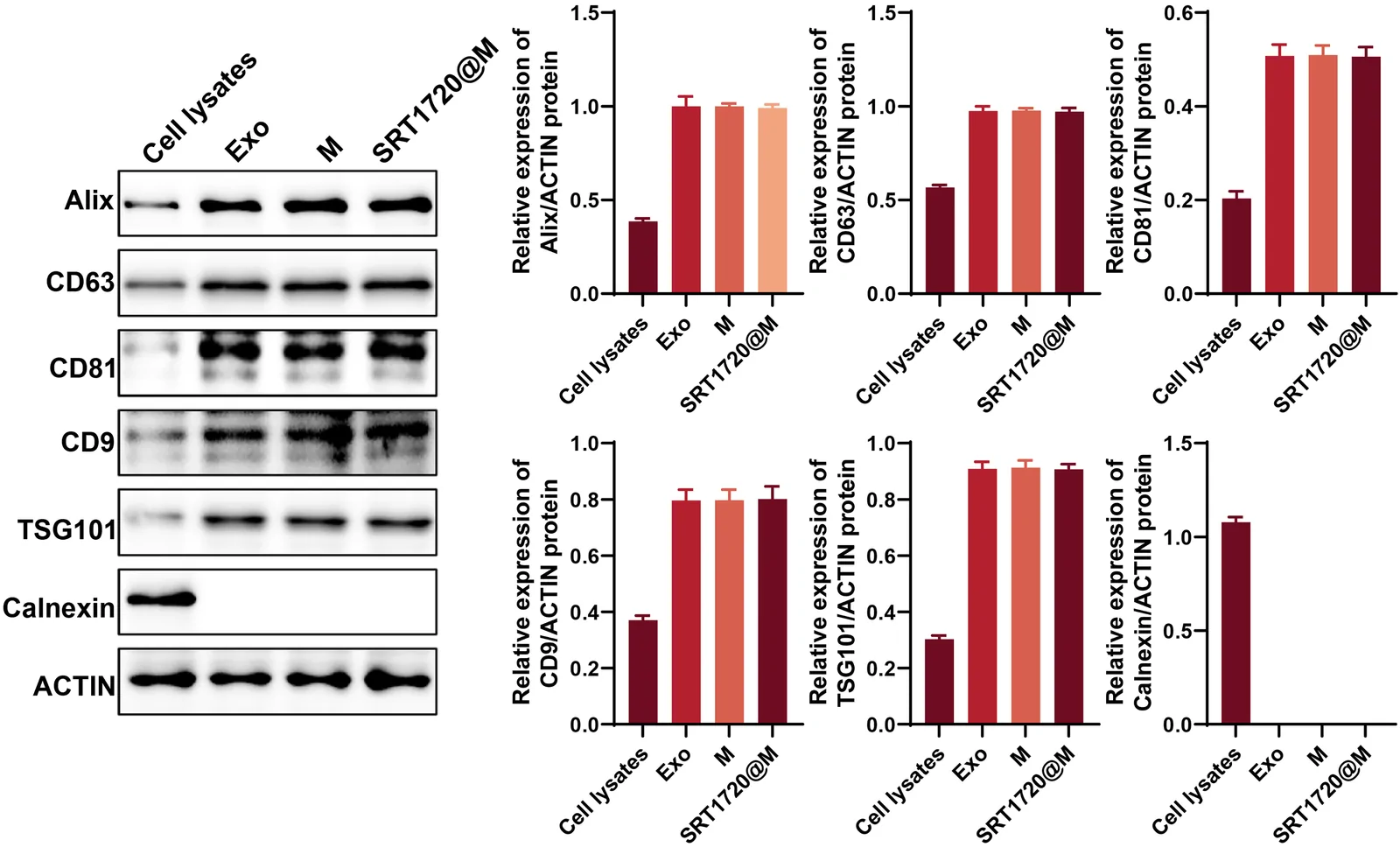
Exosome marker protein expression in M and SRT1720@M nanovesicles. (A) Representative Western blots of exosomal markers (Alix, CD63, CD81, TSG101) and the negative control Calnexin in cell lysates, natural exosomes (Exo), M, and SRT1720@M. (B) Quantitative analysis of protein expression normalized to ACTIN. Data are mean ± SD (n = 3). Note: Calnexin is an endoplasmic reticulum marker used as a negative control to exclude contamination from other cellular components.

**Fig. S15:**
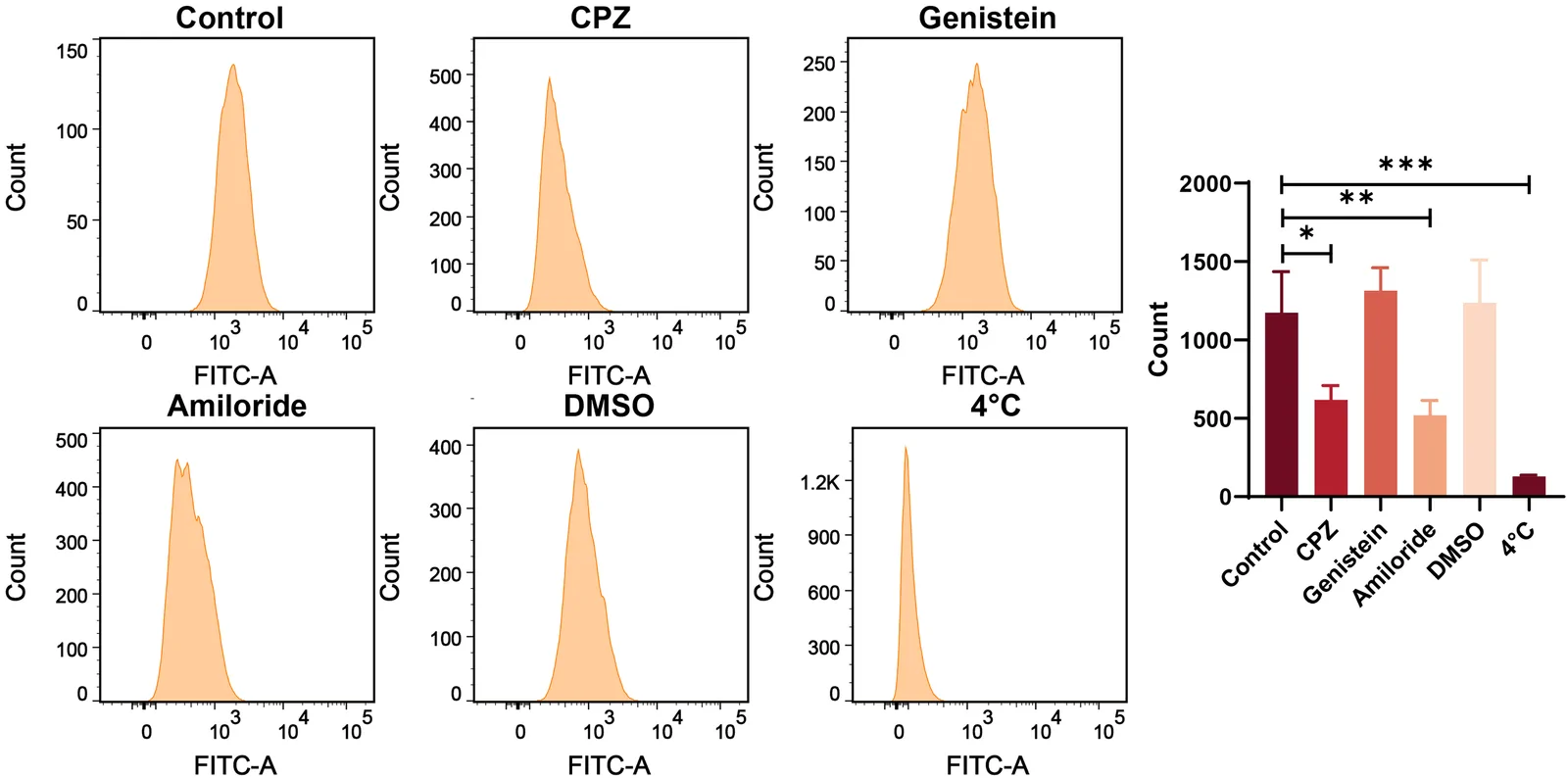
Internalization pathway of FITC-labeled SRT1720@M. (A) Representative flow cytometry histograms showing cellular uptake of FITC-SRT1720@M after pretreatment with different endocytosis inhibitors: chlorpromazine (clathrin inhibitor, 10 μg/mL, 30 min), genistein (caveolae inhibitor, 200 μM, 30 min), amiloride (macropinocytosis inhibitor, 1–2 mM, 30 min), DMSO (solvent control), and low temperature (4 °C, 60 min). (B) Quantification of mean fluorescence intensity (MFI) from three independent experiments. Data are mean ± SD (n = 3). **P < 0.01 vs. control; n.s. indicates not significant.

**Fig. S16:**
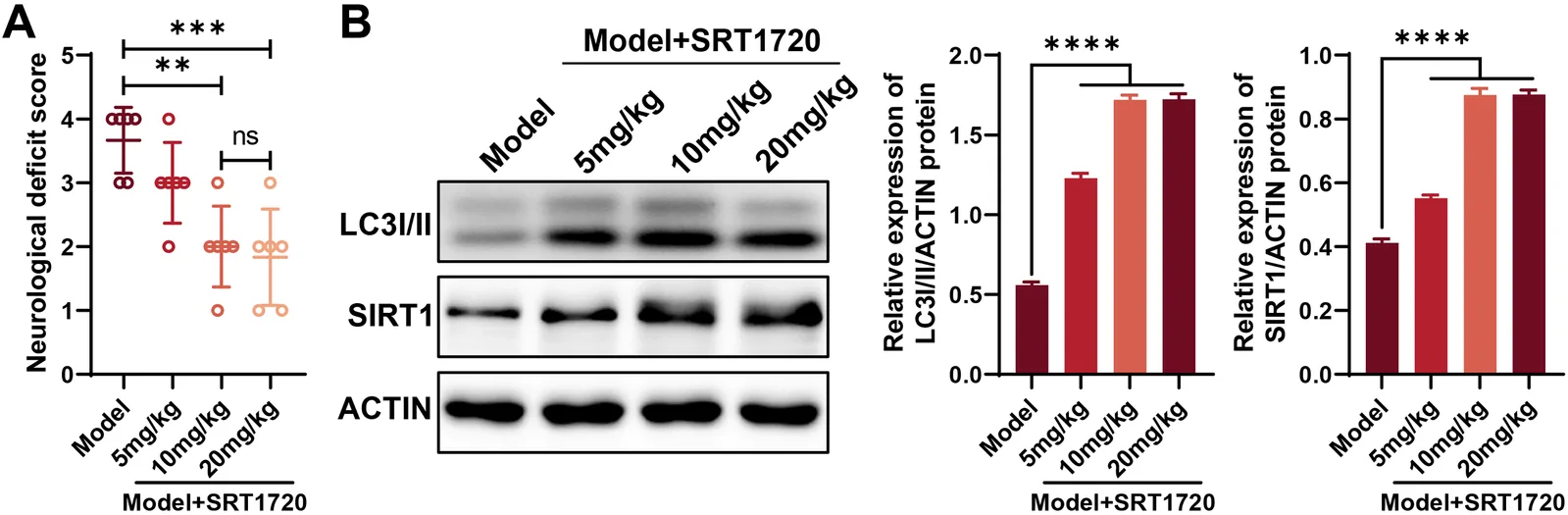
Dose-ranging study of SRT1720@M in DCI rats. (A) Neurological deficit scores (NDS) at 24 h after the last dose (n = 6 per group). (B) Representative Western blots and quantification of SIRT1 and LC3-II/I expression in brain tissues (n = 3). Data are mean ± SD. *P < 0.05, **P < 0.01, ***P < 0.001 vs. Model; n.s. indicates not significant between 10 and 20 mg/kg groups.

**Fig. S17:**
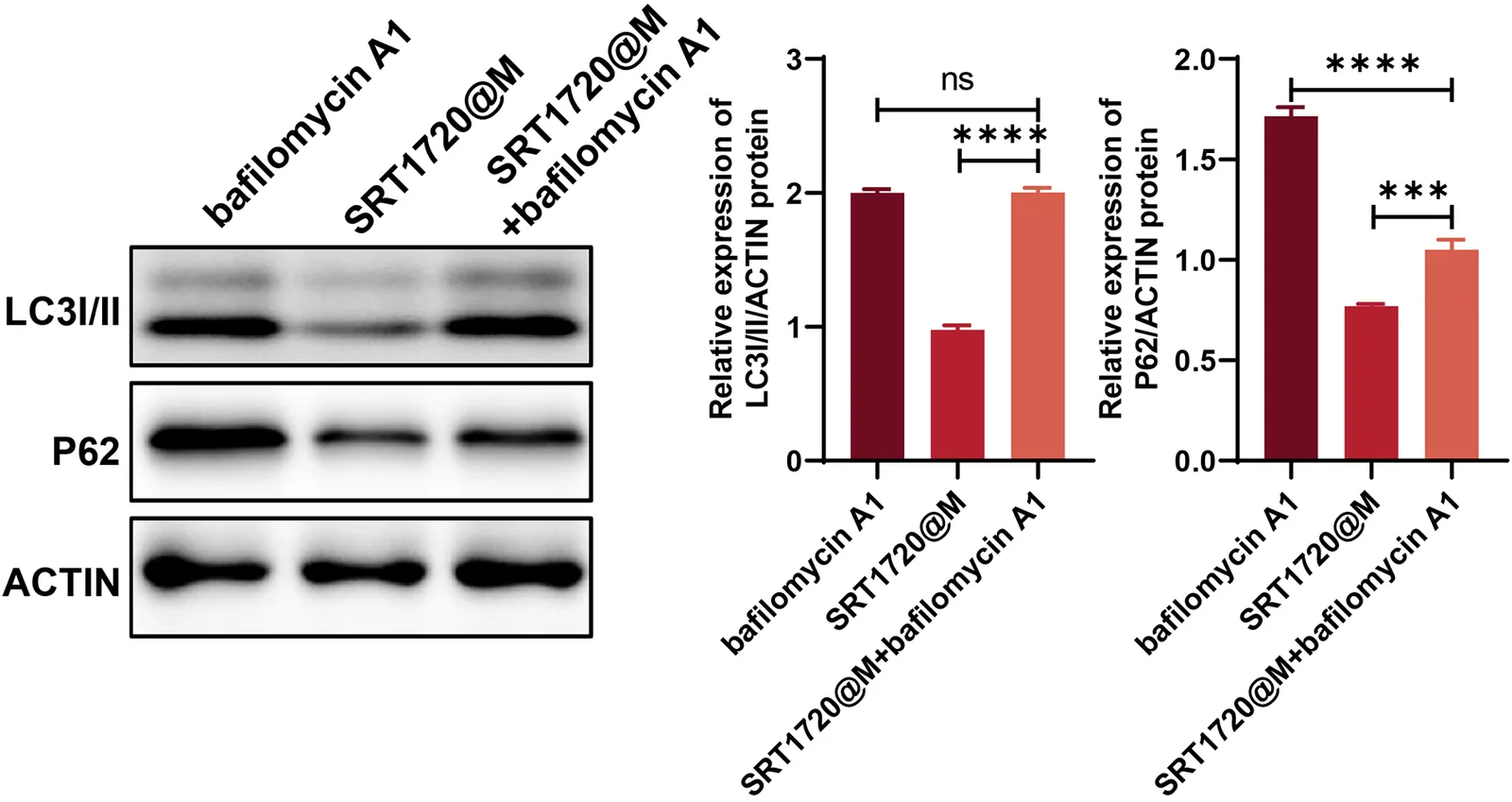
Autophagic flux analysis of SRT1720@M in the OGD/R + HG model. (A) Representative Western blots of LC3-I/II and p62 under control conditions or after bafilomycin A1 (baf A1, 100 nM, 4 h) treatment in model and SRT1720@M groups. (B) Quantification of LC3-II/I ratio and p62 levels normalized to ACTIN. Data are mean ± SD (n = 3). **P < 0.01 vs. Model without baf A1; n.s. indicates not significant.

**Fig. S18:**
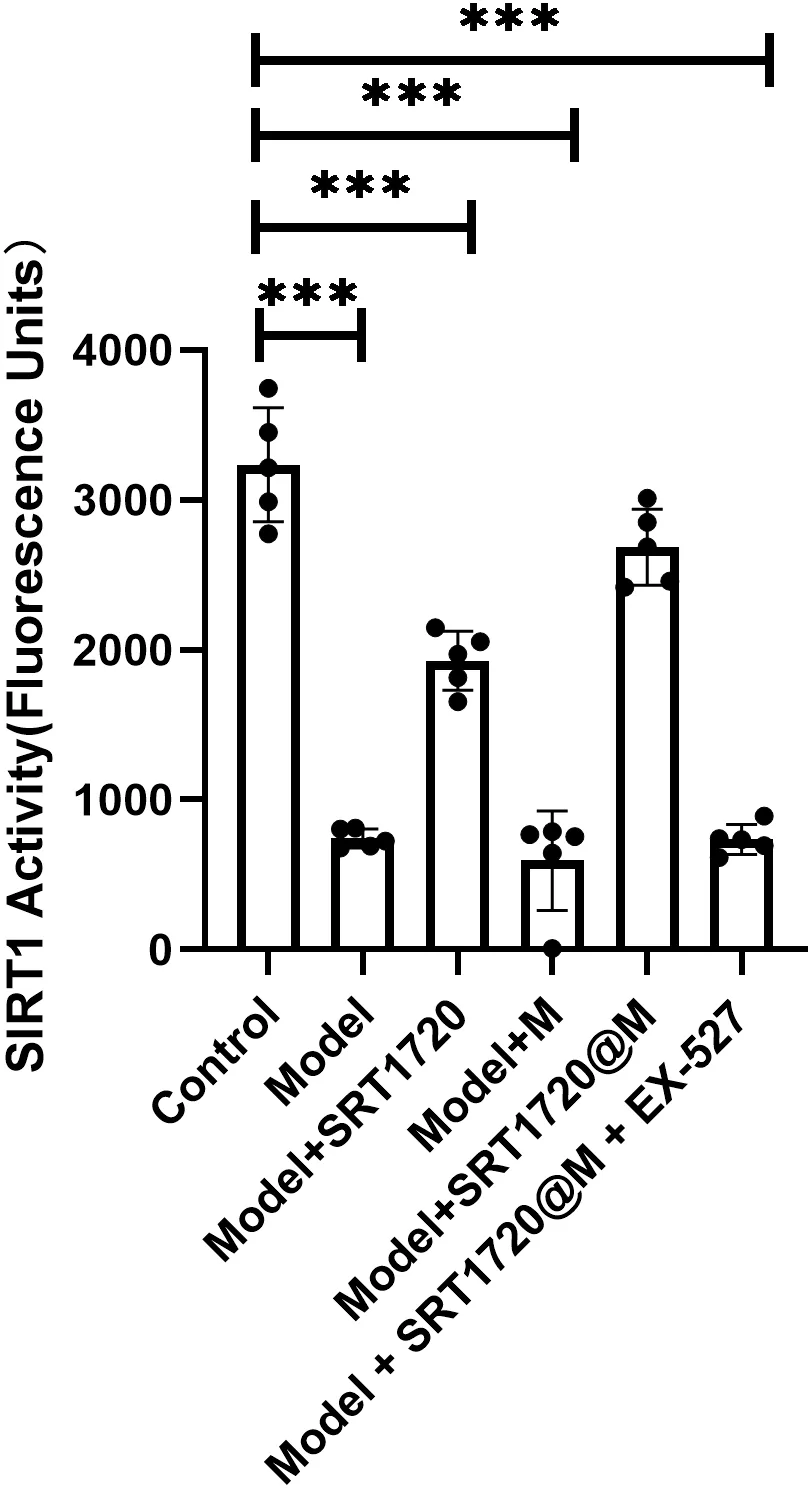
SIRT1 enzymatic activity in primary hippocampal neurons. Data are mean ± SD (n = 3). ***P < 0.001 vs. Control;

## CRediT authorship contribution statement

**Qifeng Huang:** Writing – original draft, Data curation. **Fang Zhao:** Validation, Methodology, Data curation. **Bo Sun:** Writing – review & editing, Writing – original draft, Data curation. **Xie Yang:** Validation, Data curation. **Miao Chen:** Writing – review & editing, Supervision, Project administration.

## Consent for publication

Not applicable.

## Ethical approval and consent to participate

The Ethics Committee of Hainan Medical University approved all animal procedures.

## Funding

This study was supported by (1) Hainan Province Clinical Medical Center and (2) Science and Technology Special Fund of Hainan Province, No. ZDYF2025SHFZ048. (3) 10.13039/501100001809National Natural Science Foundation of China (Reginal Science Foundation), No. 82560389; and (4) Discipline Construction Project of Shanghai Pudong New Area Health Commission (Key Discipline Group), No. PWZxq2022-12.

## Declaration of competing interest

The authors have no relevant financial or non-financial interests to disclose.

## Data Availability

The data used and/or analyzed during the current study are available from the corresponding author upon reasonable request.
